# Small molecule electro-optical binding assay using nanopores

**DOI:** 10.1038/s41467-019-09476-4

**Published:** 2019-04-17

**Authors:** Shenglin Cai, Jasmine Y. Y. Sze, Aleksandar P. Ivanov, Joshua B. Edel

**Affiliations:** 1 Department of Chemistry, Imperial College London, Exhibition Road, London, SW7 2AZ UK; 2 Precision Medicine Laboratories, Precision Medicine & Genomics, IMED Biotech Unit, AstraZeneca, Cambridge, CB4 0WG UK

**Keywords:** Single-molecule biophysics, Bioanalytical chemistry, Fluorescent probes, Sensors

## Abstract

The identification of short nucleic acids and proteins at the single molecule level is a major driving force for the development of novel detection strategies. Nanopore sensing has been gaining in prominence due to its label-free operation and single molecule sensitivity. However, it remains challenging to detect small molecules selectively. Here we propose to combine the electrical sensing modality of a nanopore with fluorescence-based detection. Selectivity is achieved by grafting either molecular beacons, complementary DNA, or proteins to a DNA molecular carrier. We show that the fraction of synchronised events between the electrical and optical channels, can be used to perform single molecule binding assays without the need to directly label the analyte. Such a strategy can be used to detect targets in complex biological fluids such as human serum and urine. Future optimisation of this technology may enable novel assays for quantitative protein detection as well as gene mutation analysis with applications in next-generation clinical sample analysis.

## Abstract (web summary)

Nanopore detection of small molecules can be improved using molecular carriers, but separating a small analyte from the carrier signal can be challenging. Here the authors address this challenge using simultaneous electrical and optical readout in nanopore sensing to detect small molecules and quantify binding affinities.

## Introduction

The development of analytical tools for early-stage diagnosis of diseases can often be limited by the low-abundance of particular biomarkers. This is compounded by the complexity of probing analytes within biological fluids. At present, the most widely used commercial technologies for detection of given molecular targets are based on immunoassays or gel electrophoresis. However, these methods tend to lack the required sensitivity especially when one needs to probe low biomarker concentrations in complex fluids such as serum. While electrochemical methods have the potential to address some of these limitations, they cannot currently be used in the presence of a large number of similar molecules, thus often failing to reveal the presence of distinct molecular signatures that can mark the early stages of disease onset. Single-molecule methods have a clear advantage in the sense that biomarkers can be probed one at a time at relevant concentrations rather than ensemble averaged. A number of different strategies have been adopted, with fluorescence^1,2^, nanopores^3^, and field-effect transistors^4–6^ being widely used.

For example, nanopores are a class of single-molecule sensors used for the detection of analytes such as DNA, RNA and proteins^3,7^. In a typical experiment, molecules are electrokinetically translocated by an externally applied electric field through a nanopore usually less than 30 nm in diameter. The analytes are translocated through the nanopore one at a time, resulting in characteristic temporal modulation of the measured ionic (nanopore) current. From these modulations, one can extract information on molecular properties such as size, composition, and interactions with other biomolecules. However, this method generally lacks sufficient selectivity and sensitivity, especially for molecules that are much smaller than the diameter of the nanopore. Strategies to circumvent some of these limitations include chemical modification around the pore^8,9^, reduction in pore dimensions, and use of high bandwidth amplifier^10^.

Recently, the use of molecular carriers has shown great promise to improve both selectivity and sensitivity and relies on binding biomarkers to a carrier backbone often made from DNA^11–14^. A multilevel current signal is measured, with the contribution of the bound biomarker being superimposed on the signal due to the translocated carrier. The use of DNA as a carrier has several advantages enabling efficient transport of proteins through the pore as well as enabling better control of the rate of transport. However, for this method to be successful, the corresponding biomarker must be sufficiently large to ensure the superimposed signal can be distinguished from that of the carrier. Furthermore, it is not always trivial to distinguish between the signal arising from a target analyte bound to the carrier and a carrier that translocates in a folded state.

To address these challenges, the advantages of both nanopore sensing and single-molecule fluorescence spectroscopy can be combined to enable an efficient strategy for small molecule detection using nanopores. For example, fluorescent probes can be used to target molecules that are difficult to detect using conventional nanopore sensing, while the combined electrical and optical signals can be used to quantify binding affinities, as well as to selectively confirm the presence of a particular biomarker.

A number of groups, including our own, have already demonstrated fluorescence detection coupled to nanopores^15–22^. However, these approaches generally rely on labelling of the target molecule, limiting their applicability. We explore the possibility of using simultaneous detection without the need for covalently labelling the target analyte and validate the feasibility of this strategy by detecting the presence and binding of small DNA oligomers and proteins  to the carriers with single-fluorophore sensitivity. Importantly by attaching the target to a carrier, the molecule spends more time within the detection volume resulting in increased sensing times and as a result, improved signal to noise ratio. Furthermore, an important advantage is that the nanopore is perfectly aligned with the intensity maximum of the diffraction limited fluorescence detection volume resulting in improved photon count rates.

**Fig. 1:**
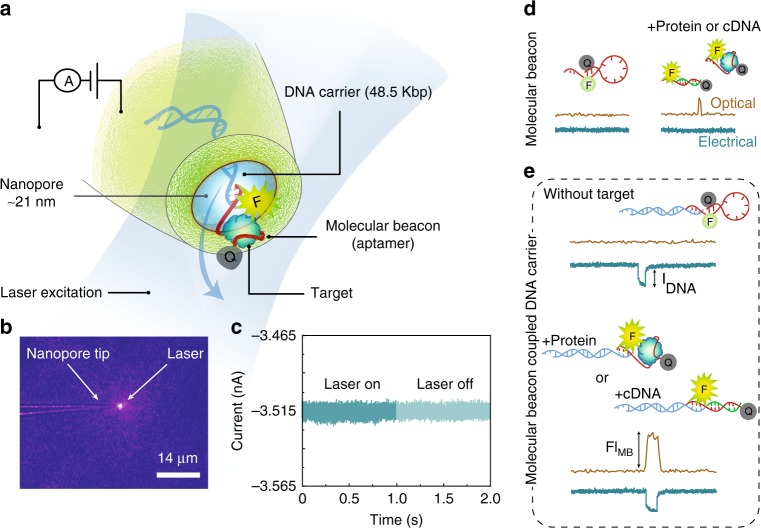
Conceptual overview of the experimental approach. **a** Schematic of the electro-optical configuration where a nanopore is integrated with a single-molecule fluorescence confocal microscope. Upon the application of the electric field, individual DNA carriers translocate through the nanopore. The optical detection volume is superimposed on the nanopore, and both the ionic current and fluorescence intensity time trace are obtained in a synchronised manner. **b** Optical image showing laser illumination at the tip of the nanopore (21 ± 2 nm, mean ± standard deviation). **c** Noise characteristics with the laser illumination switched ON (laser power 198 ± 6 μW) and OFF, respectively. **d** A molecular beacon (MB) without a carrier produces no signal in the electrical channel and a low optical signal when bound to a target. **e** MBs are hybridised to a DNA carrier for the single-molecule detection of small oligonucleotides or proteins. Without target binding, the signal is only observed in the electrical detection channel. When bound to complementary DNA (cDNA) or protein, a synchronised signal is observed in both channels due to the opening of the MB and the increased distance between the fluorophore and the quencher probes. Fluorescence signals obtained are higher when the MB is bound to the carrier due to the increase in residence time as well as excellent alignment with the intensity maximum of the diffraction limited laser beam

The fraction of synchronised events between the electrical and optical channels can be used to quantify the presence of a target as well as the concentration. For example, molecular beacons (MBs) can be designed and incorporated into the DNA carrier to screen for small proteins and complementary DNA sequences (Fig. 1). MBs are short oligonucleotide fluorophore/quencher probes containing a “stem-loop” structure, whose sequences can be designed as needed for a range of binding targets.^23,24^. The MB remains in its quenched state until the target analyte binds after which the fluorescence will then be restored. Use of MBs are exceptionally advantageous as the target molecule does not have to be directly fluorescently labelled. In this manuscript, the  MBs were designed to incorporate  aptamer sequences such that the corresponding protein will unravel the MB (Fig. 1d, e).

## Results

### Validation of synchronised detection

Simultaneous electro-optical measurements require precise alignment between the nanopore and the diffraction limited optical detection volume (*ω*_0_ = 450 ± 5 nm, mean ± standard deviation)^19,21^, as shown in Fig. 1a and Supplementary Figs. 1 and 2. For this, we used quartz nanopipettes, a subclass of nanopores, as this is an ideal platform to use due to ease of operation and no detectable autofluorescence, unlike more common materials such as SiN_*x*_^19,20^. Alignment was achieved by mounting the nanopipette on a coverslip inserted into a custom sample holder on a high-resolution motorised stage (with a 10 nm step size) and using an electron multiplying charge coupled device (emCCD) camera (Fig. 1b) to visually align the *x*−*y* axes. The *z* height was fine-tuned by scanning this axis in 10 nm step sizes until scattering was observed using an avalanche photodiode (APD).

The nanopipettes were fabricated using protocols previously reported by our group^11,25,26^, yielding an average pore size of 21 ± 2 nm (*n* = 20), as measured by scanning electron microscopy (SEM, Supplementary Fig. 3). The nanopore conductance was found to be 3.7 ± 0.2 nS using 0.1 M KCl (Supplementary Fig. 4). Previously it has been shown that laser illumination severely affects electrical noise characteristics due to photo-induced heating of the electrolyte and changes in surface charge on the pore surface^21,22^. In our system, we observed almost no additional electrical noise under 488 nm, 198 ± 6 μW laser exposure as can be seen by the lack of measurable increase in the baseline ionic current (Fig. 1c), and no significant change in the power spectral densities (Supplementary Fig. 5).

**Fig. 2:**
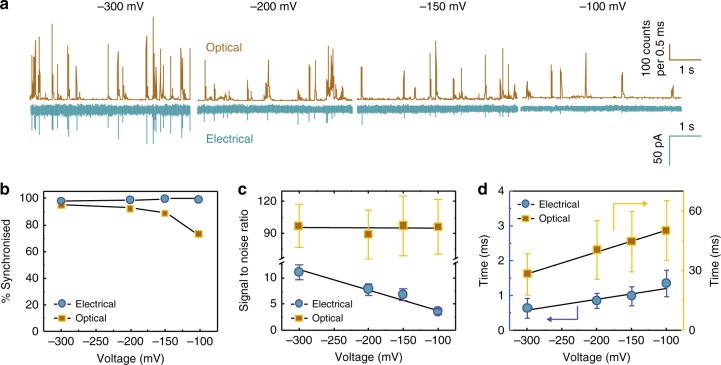
Single-molecule electro-optical detection of 5 kbp DNA labelled with YOYO-1. **a** Photon and current time traces for the translocation of 100 pM 5 kbp DNA-YOYO-1 in 100 mM KCl, 10 mM Tris-HCl, 1 mM ethylenediaminetetraacetic acid (EDTA) buffer (pH = 8). The resampling time for the photon time trace is 500 μs and the filter cut-off frequency for the current time trace is 10 kHz. Laser power was 90 ± 3 μW. **b** Percent synchronisation, **c** signal to noise and **d** dwell time as a function of applied voltage for the electrical (blue circles) and optical (brown squares) channels respectively. The error bars represent the accumulation of statistics from at least three different nanopipettes

To confirm appropriate alignment, YOYO-1 fluorescently labelled 5 kbp DNA was used to monitor the percentage of synchronised events between the optical and electrical channels (Fig. 2). The percent synchronisation was also quantified as a function of positional offset between the confocal volume and nanopipette tip (Supplementary Fig. 6). The DNA was translocated through the pipette using voltages ranging from −300 to −100 mV (Fig. 2a) and −80 to −40 mV (Supplementary Fig. 7). The percentage of electrically synchronised events was very high, indicating excellent alignment with *S* = 98.4% (*n* = 249), *S* = 98.9% (*n* = 174), *S* = 100% (*n* = 144) and *S* = 100% (*n* = 158) for −300, −200, −150 and −100 mV, respectively (Fig. 2b). Notably, these values are significantly higher than the synchronisation levels that we have previously reported (92.7%)^21^. It should be noted that a small underlying fraction of events appeared solely in the optical channel with much lower average intensities (Supplementary Fig. 8). These events are likely due to molecules freely diffusing around or near the optical probe volume without being translocated through the nanopore^18^. The level of synchronisation further decreased at lower voltages (*S* = 71.8%, *n* = 220 at −100 mV) in large part due to the lower peak amplitude of the electrical events causing them to be embedded within the noise. For example, the signal-to-noise (S/N) ratio in the electrical detection channel decreased from 11 ± 1.4 to 3.6 ± 0.5 for −300 to −100 mV (Fig. 2c and Supplementary Fig. 9). The optical peak amplitude was not dependent on voltage and hence remained constant (S/N = 94.5 ± 3.9 across all voltages) (Fig. 2c and Supplementary Fig. 10). Another interesting observation was in the comparison between translocation times recorded in both channels (Fig. 2d and Supplementary Fig. 11). As expected, the times decreased as a function of increasing voltage; however, optical events were typically over one order of magnitude slower. This was due to the signal being a convolution of both the translocation process and free diffusion of the molecule within the probe volume. This is consistent with what we have observed previously^19,21^.

### Single-fluorophore sensitivity

To truly take advantage of using coincident electro-optical detection, the sensitivity was quantified at the single-fluorophore limit. A λ-DNA carrier with a 12-base overhang was used to hybridise a complementary strand (labelled with a single atto 488 dye) on the 3′ end (Supplementary Fig. 12). The overhang enables facile hybridisation with any probe that can be used to selectively target and bind to an analyte^11^. In this context, a simultaneous detection strategy is useful as the nanopore effectively acts as a physical gate to deliver and ‘read’ the DNA carriers, whereas the optical signal can be used to report on binding with target biomolecules, including ones that are much smaller than the pore dimensions.

**Fig. 3:**
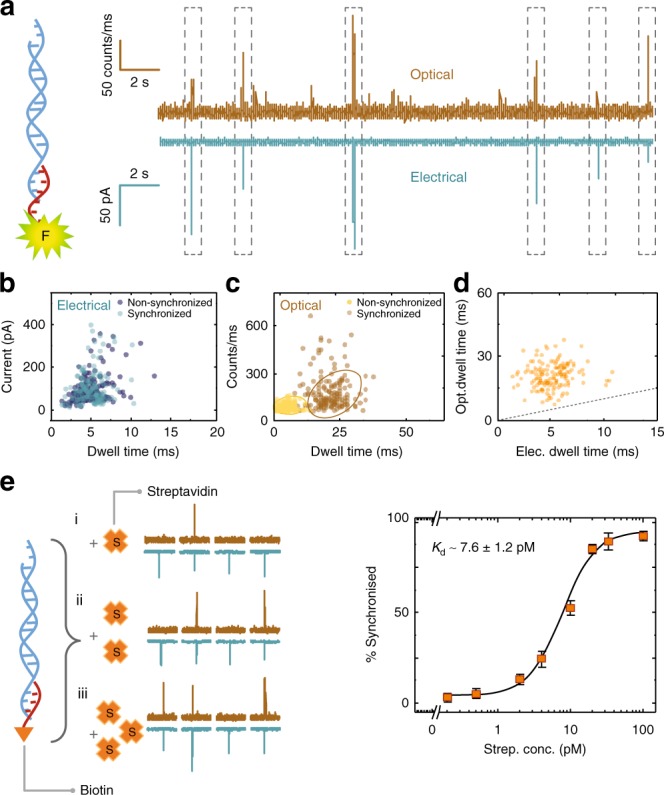
Single-molecule and single-fluorophore sensitivity. **a** Photon and current time traces for the translocation of λ-DNA carriers  bound to fluorescently labelled DNA oligonucleotides (5′-AGGTCGCCGCCC GGTTGGGTGGGTTGG-Atto 488-3′) in 100 mM KCl, buffer (pH = 8). **b** Scatter plots for dwell time vs. current amplitude/intensity for both electrical and **c** optical measurements (*N* = 178 non-synchronised, *N* = 152 synchronised). Data for both synchronised and non-synchronised events are shown. **d** Scatter plot showing synchronised optical vs. electrical dwell times. The dashed line has a slope equal to 1 and represents the ideal case of both optical and electrical events having the same dwell times. **e** A binding assay was demonstrated using a DNA carrier modified with a biotinylated oligonucleotide which can then bind to streptavidin. Translocation experiments were performed at −300 mV bias in 100 mM KCl buffer (pH = 8). A final DNA carrier concentration of 10 pM was used and incubated with Dylight 488-conjugated streptavidin at varying concentrations at room temperature. Error bars indicate the standard deviation for data obtained from three different nanopipettes. In all cases, the laser power was 198 ± 6 μW

A typical intensity-time trace for a 10 pM solution of the λ-DNA-oligonucleotide-dye complex obtained at a voltage of −300 mV is shown in Fig. 3a. Synchronised events are highlighted with a dashed box (see also Supplementary Fig. 13). Controls for both λ-DNA and the dye-oligonucleotide are shown in Supplementary Fig. 14. The majority of events were coincident with a total of 287 electrical events being detected and 267 of them being synchronised with optical channel (*S* = 93%). We attribute the remaining 7% to be most likely caused by unsuccessful hybridisation of the oligonucleotide. Both synchronised and non-synchronised electrical events had comparable dwell times and peak amplitudes. For example, at a voltage of −300 mV, synchronised events yielded a mean dwell time of 5.1 ± 1.6 ms and mean peak current of 63 ± 25 pA, while non-synchronised events yielded mean values of  5.0 ± 1.7 ms and 65 ± 33 pA respectively (Fig. 3b). These values were consistent with controls for the standard translocation of λ-DNA (Supplementary Fig. 15). However, as can be seen in Fig. 3c, the optical signal produced events which were at least fivefold longer in duration, when comparing synchronised and non-synchronised events (21.3 ± 4.6 vs. 4.4 ± 2.1 ms), see also scatter plot of synchronised optical vs. electrical dwell times, Fig. 3d. The extended dwell time is in part due to a large difference in diffusion coefficients when comparing the fluorophore bound and unbound to DNA. The oligo-carrier complex spend more time within the optical detection volume due to the carrier slowing down the transport. This is highly advantageous as freely diffusing single molecules are often photon count limited, enabling improved detection statistics. A more detailed analysis of voltage dependence on dwell time and peak amplitude/intensity is shown in Supplementary Fig. 16 where similar improvements are seen at both higher and lower voltages.

### An electro-optical single-molecule protein binding assay

The detection platform can be further extended to perform an electro-optical binding assay. A 12-mer biotinylated oligonucleotide (complementary to the 3′ end of λ-DNA) was hybridised to the carrier  (see Methods for details) for detection of streptavidin. The biotinylated carriers were incubated with fluorescently labelled streptavidin (Dylight 488) followed by translocation at a final concentration of 10 pM. As expected, the free carriers produced a signal in the electrical detection channel, streptavidin on its own in the optical channel, and the carrier-streptavidin complex in both channels (Supplementary Fig. 17). Detection of such low protein concentrations is not typical when sensing proteins natively without a carrier, due to fast translocation times and event rates often being significantly lower than predicted from the Smoluchowski rate equation, which often necessitates protein concentrations well in excess of 10s−100s nM^27^. Addition of the carrier improves the transport in part due to the negatively charged DNA backbone as well as facilitates detection of a bound event via the synchronised electro-optical signal (Fig. 3e). Binding of streptavidin to the carrier produces a substantial improvement in both the dwell time and total fluorescence intensities (Supplementary Figs 18, 19). Although previous studies^12,13,28^ have demonstrated that the binding of protein to a long DNA carrier could be identified by reading out the sub-levels of the signal, fluorescence enables the direct quantification of a bound event and eliminates any possible false positives due to the influence on DNA conformational changes such as folds or knots^29,30^. For example, using our nanopore configuration, 32−36% of all events produced sub-peaks for both λ-DNA on its own and the biotinylated carrier. In contrast, 0% synchronisation was observed in the optical channel producing no false positives (Supplementary Fig. 20).

The binding affinity can be determined from the synchronised fraction (the percentage of synchronised counts over all electrical counts) as a function of the streptavidin concentration (Fig. 3e). As expected, the fraction of synchronised events raises with increasing streptavidin concentration. In this case, the carrier concentration was kept constant at 10 pM, and streptavidin concentration was ramped from 0 to 100 pM. At 0 pM streptavidin, only events in the electrical channel were observed while at a two fold excess the synchronised fraction (85.7 ± 2.2%) increased accordingly and reached a plateau representing the saturation of streptavidin bound to the biotinylated carrier. The Hill binding model, which typically describes the equilibrium state of reversible molecular binding^31^, could then be used to determine the apparent dissociation constant. Using this approach, a binding affinity of *K*_d_ = 7.6 ± 1.2 pM was obtained. This *K*_d_ value is roughly two orders of magnitude larger than that measured for wild-type streptavidin-biotin (*K*_d_ = 10^−14^ M)^32^ and comparable (*K*_d_ = 10^−11^ M)^33^ to cases where the association may be affected by dye conjugation^34^. Attachment of biotin moieties to a larger group can also restrict its free diffusion and thus reduce the binding affinity^35^.

### Molecular beacon-modified DNA carriers

**Fig. 4:**
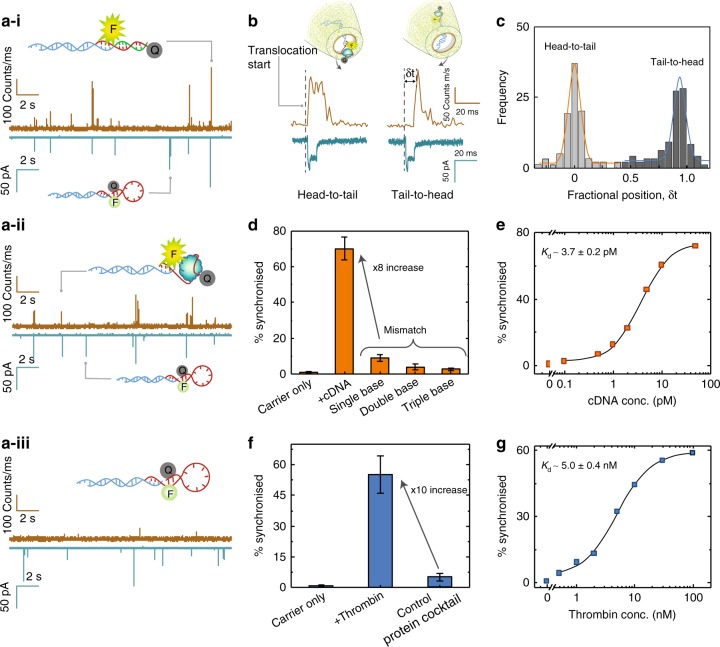
Detection of DNA oligos and proteins using molecular beacons. Photon and current time traces are shown for the translocation of **a-i** DNA MB-Carrier-cDNA, **a**-**ii** DNA MB-Carrier-Thrombin, and **a-iii** DNA MB-Carrier. The DNA carrier, cDNA, and thrombin concentrations were 10 pM, 50 pM, and 30 nM respectively. Translocations were recorded at −300 mV bias in a buffer of 100 mM KCl (pH = 8). **b** It was possible to determine the orientation of the complex translocating through the nanopore by characterising the **c** fractional position for the onset of the optical signal relative to the onset of the electrical signal (*N* = 193). **d** Percent synchronisation between the optical and electrical channels for cDNA bound to the MB-Carrier along with controls including single, double and triple base mismatches. **e** A binding affinity of 3.7 pM was calculated by fitting a Hill binding model for cDNA as a function of % synchronisation. **f** Percent synchronisation between the optical and electrical channels for thrombin bound to the MB-Carrier along with controls. **g** A binding affinity of 5.0 nM was calculated by fitting a Hill binding model for thrombin as a function of % synchronisation which was in agreement with existing bulk methods. Error bars in **f** and **h** were determined using data obtained from a minimum of three different nanopipettes. In all cases, the laser power was 198 ± 6 μW

While the detection and quantification of small biomolecules was demonstrated, biotin-streptavidin binding is to a large extent well established. Furthermore, the direct labelling of the sample can be time-consuming and complex, which restricts the range of targets that can be probed. With this in mind, we designed molecular carriers that integrate MBs and are ideally suited for “label-free” sensing applications. MBs are short oligonucleotides with a stem-loop structure, whose sequences could be designed as needed to specifically recognise a range of nucleic acids via hybridisation or proteins using aptamer sequences^23,36,37^. Instead of direct labelling of the targets, the fluorophore−quencher pair was incorporated into the MB-Carrier. Fluorescence could then be restored upon binding to a target, as shown in Fig. 4a and Supplementary Figs 21–22. As a control translocation of the MB without the carrier present produced no electrical events and minimal optical events all with very low intensity (Supplementary Fig. 23).

As an example, we incorporated a 15 mer thrombin-binding aptamer (TBA)^38^ into the loop of the MB. TBA was selected due to its well-established structure, and high affinity towards thrombin^38–40^, the design principle and sequence of this TBA-embedded MB and its incorporation into the λ-DNA to form an MB-Carrier are described in detail in the Methods section and Supplementary Fig. 24. Three groups of experiments were performed: (i) MB-Carrier (10 pM) in the presence of a strand complementary to the TBA aptamer (15 mer: 5ʹ-CCA ACC ACA CCA ACC-3′, 50 pM), (ii) MB-Carrier (10 pM) in the presence of thrombin (30 nM), and (iii) MB-Carrier (10 pM) on its own (control experiment) (Fig. 4a). For DNA hybridisation-based experiments, the MB loop undergoes structural transition when bound with target DNA to form a duplex state, resulting in the separation of the fluorophore from the quencher. For thrombin binding, the MB aptamer changes from its stem-loop shape to form a G-quadruplex structure upon binding, extending the distance between the fluorophore and the quencher^36^.

Two classes of events were observed with either the electrical signal coming first or alternatively the combined electro-optical  signals originating at approximately the same time (Fig. 4b and Supplementary Fig. 25). This correlates to the two different orientations in which the complex is transported through the pore. In the “head to tail” configuration, the MB is transported through the pore first, and conversely, in the “tail to head” configuration, the carrier is transported first. To quantify the translocation orientation, we normalised the electrical dwell times with the start time being defined as 0 and the end time as 1. The fractional position of the optical signal relative to the electrical could then be plotted (Fig. 4c). Two populations could be observed, one at 0.003 ± 0.1 and the other at 0.930 ± 0.1 which corresponds to the two possible orientations. A similar trend was also observed when binding the MB-Carrier to cDNA (Supplementary Fig. 26). Determining orientation is often difficult purely based on electrical data. However, an electro-optical approach facilitates the extraction of this information. 

Much like the case for streptavidin, binding affinities and selectivity could be determined (Fig. 4d, e). For example, the selectivity of the MB-Carrier was compared to a corresponding complementary DNA strand (cDNA, 15 bases) containing single, double, and triple base mismatches (Fig. 4d and Supplementary Fig. 27). Increasing the number of mismatches in the DNA sequence destabilises the DNA duplex. We used 2−4 base spacing when introducing mismatches, which results in a significant decrease in DNA melting temperature and hence lower probability of forming a stable duplex^41^. We expected the latter to be manifested in a decrease in the number of synchronised events, with relative conservation of the mean peak intensity and dwell times. *S* = 70.3 ± 6.4%, for cDNA, and *S* = 8.9 ± 1.92%, 3.9 ± 1.72%, and 2.7 ± 0.61% for single, double and triple base mismatch, respectively. The eightfold improvement in signal for the cDNA as compared to the single base mismatch  highlights the excellent capability of this approach to selectively discriminate single nucleotide polymorphism in individual molecules, without the need for amplification. By performing a titration and fitting using the Hill model (Fig. 4e), the binding affinity was determined to be 3.7 ± 0.2 pM for cDNA which is close to the value estimated from the Gibbs free energy (0.9 pM) (see Supplementary Note 1). The detection limit was determined to be 0.1 pM, based on comparing the synchronised fraction to the blank control (0.8 ± 0.4%). It should be noted that this limit is lower than more conventional single-molecule counting methods (0.7 pM)^42^ including two-colour coincident detection (0.5 pM)^43^.

As a TBA sequence was incorporated in the MB, a similar experiment could be performed with the addition of protein (Fig. 4f). The thrombin selectivity was quantified  by performing control experiments within a much more concentrated background (>300-fold excess for each target) containing a mixture of proteins including lysozyme, trypsin, α-synuclein, and insulin (Fig. 4f and Supplementary Fig. 28). Importantly a tenfold increase in the percent synchronisation could be observed when comparing thrombin to the protein mixture which highlights the excellent selectivity and possibility to discriminate between the target protein and other proteins. The binding affinity (Fig. 4g) was determined to be 5.0 ± 0.4 nM, which is in excellent agreement with the values measured (4.87 to 10 nM) using alternative approaches^36,37,44^ (see also Supplementary Fig. 29). In addition, the detection limit for thrombin was determined to be 0.5 nM, which is also significantly lower than other reported methods^11,45^.

**Fig. 5:**
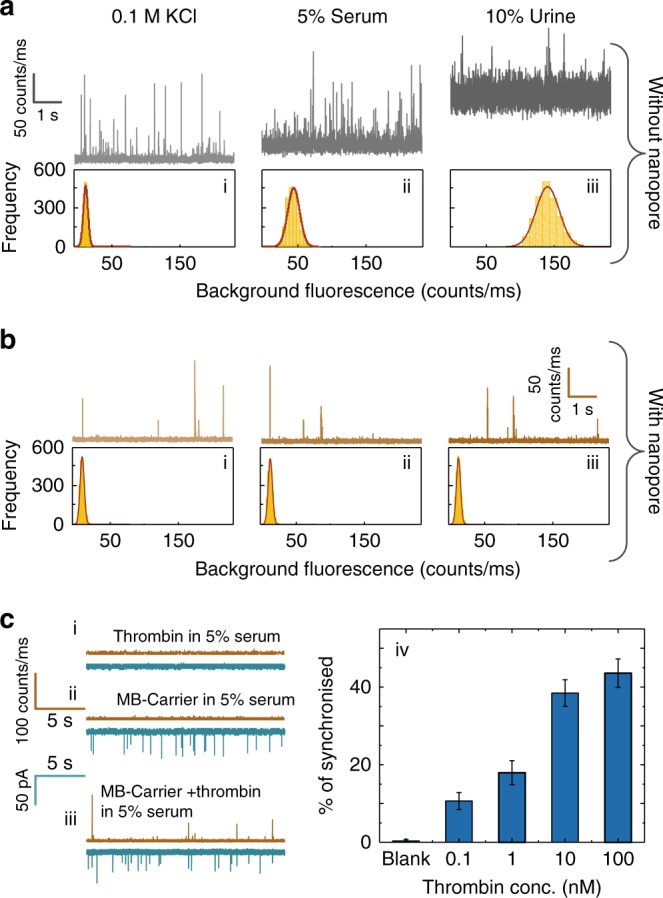
Sensing of cDNA and protein targets in human serum and urine. **a** Photon time traces for the detection of cDNA bound to the MB-Carrier in (i) 0.1 M KCl (pH = 8), (ii) 0.1 M KCl + 5% human serum, (iii) and 0.1 M KCl + 10% urine. Conventional confocal single-molecule methods were used (i.e. droplet on a coverslip). **b** Comparable traces to those shown in (**a**) using a nanopore. A significant decrease in background fluorescence is observed in part due to the solution being confined to inside the nanopipette. The reservoir outside the nanopipette only contains a 0.1 M KCl buffer solution. Translocation experiments were performed at −300 mV and in all cases, the laser power was 193 ± 6 μW. **c** Photon and current time traces are shown for the translocation of (i) thrombin in 5% serum, (ii) MB-Carrier in 5% serum, and (iii) MB-Carrier bound to thrombin in 5% serum. The MB-Carrier and thrombin concentration was 30 pM and 1 nM respectively. (iv) Percent synchronisation between the optical and electrical channels for thrombin bound to the MB-Carrier at concentrations ranging from 0.1 to 100 nM. Error bars indicate the standard deviation for data obtained from three different nanopipettes

When using a conventional single-molecule confocal fluorescence strategy (e.g. droplet on coverslip) (Fig. 5a) for detection of cDNA bound to the MB-Carrier in complex biological fluids such as serum and urine, the background fluorescence clearly increases. However, when using a nanopore (Fig. 5b), the background fluorescence is almost identical to that of measurements taken in 0.1 M KCl. This is due to the sample being confined to within the nanopipette, the solution outside the nanopipette only consists of the KCl buffer. As can be seen this results in a substantial increase in signal to noise. An example of a binding assay performed in serum is shown in Fig. 5c and Supplementary Fig. 30; the MB-Carrier concentration was 30 pM, and thrombin was increased from 0.1 to 100 nM respectively and results are comparable to those obtained in 0.1 M KCl. An analogous study has also been performed in urine (Supplementary Fig. 31).

## Discussion

We demonstrated a selective, single-molecule binding assay using molecular carriers and molecular beacons for the detection of short oligonucleotides and single proteins in a synchronised nanopore platform. The implementation of simultaneous electro-optical detection provides a simple way of detecting molecules much smaller than the size of the nanopore without degradation of the signal-to-noise ratio. The synchronised electro-optical detection can also help to differentiate between bound target and false positives, such as fold or knot in the DNA carrier, and hence, address one of the major limitations with the use of carriers in nanopore platforms.

Using synchronised detection and molecular carriers that integrate aptamers and molecular beacons, we were able to discriminate single nucleotide mismatch (with a limit of detection as low as 0.1 pM), as well as a single protein from a protein mixture. Importantly, by quantifying the fraction of synchronised events, the platform allowed us to perform single-molecule binding assays using electro-optical detection. The probability of having a false positive show up as an apparent synchronised event is exceptionally low. Based on counting statistics, a “random coincident” event rate *R* is given by the formula *R*_random_ = 2*r*_1_*r*_2_*T*, where *r*_1_ and *r*_2_ are the events rates in the optical and electrical channels respectively and *T* is the average event times. In our case *R*_random_ ≪ 0.001 making false positives unlikely.

Potential applications such as detection of mismatched duplexes, point mutations in DNA oligonucleotides and DNA-protein complexes can be foreseen. Due to the increasing availability of aptamer sequences, a greater range of targets can be addressed by integrating relevant specific aptamers into the carriers. In principle, this combined detection strategy could be further extended in a multiplexed sensing platform by introducing different binding probes at specific sites of the carrier. This, in turn, may allow for a multiplexed capability to screen an array of molecules in complex samples with higher sensitivity and selectivity.

## Methods

### Chemicals and materials

Both 5 kbp double-stranded DNA (dsDNA) and λ-DNA (48.5 kbp) with a stock concentration of 500 μg ml^−1^ were obtained commercially from New England Biolabs. All the other DNA oligonucleotides or molecular beacon probes were synthesised by Integrated DNA Technology (sequences are shown in Supplementary Information). Streptavidin conjugated with Dylight^TM^ 488 was purchased from Thermo Scientific with a stock concentration of 1 mg ml^−1^. α-thrombin was purchased from Cambridge Biosciences, UK. The fluorescent dye, YOYO-1 (1 mM in DMSO), was obtained from Life Technology. The stock 5 kbp dsDNA (154 nM) was mixed with YOYO-1 at a ratio of 7.5 base pairs to 1 dye and incubated for 30 min prior to use^21^. Oligo sequences are available in Supplementary Methods.

### Preparation of DNA carriers

DNA carriers used in this work were designed by hybridising of λ-DNA with either a biotinylated DNA probe or an MB (see Supplementary Information for sequences). Briefly, DNA oligonucleotides were firstly diluted from a stock concentration (100 μM) using a binding buffer (140 mM NaCl, 20 mM MgCl_2_, 10 mM Tris-EDTA buffer, pH = 8.0) to 1.58 μM. Twenty-five microliters of this resultant oligonucleotide solution was then mixed with 25 μl stock λ-DNA solution and 50 μl binding buffer to achieve a total volume of 100 μl and a ratio of 1:100 of (λ-DNA: oligonucleotides). The hybridisation was then conducted by heating to 95 °C for 5 min, cooling down to 75 °C for 10 min and annealing to 25 °C at a rate of 1 °Cmin^−1^ for 90 min in total. The DNA carriers were then purified by removing the excess of oligonucleotide probes with the use of a 100 kDa MWCO Amicon Ultra Filter (Millipore, UK). This procedure included six cycles of centrifuging for 6 min at 14,000 × *g* with TE buffer (10 mM Tris-EDTA buffer, pH = 8) and recovery by centrifuging at 1000 × *g* for 2 min with turning the filter upside down. The concentration of obtained DNA carriers was determined by measuring the UV−Vis absorbance at 260 nm with a Nanodrop device (Thermo Scientific).

The MB-Carrier embedded with TBA was designed as described in the Supplementary Methods.  A 10 pM of MB-Carrier concentration was used in most of the experiments.

### Fabrication of the nanopipettes

Glass nanopipettes were fabricated from quartz capillaries (World Precision Instruments) as protocol reported by our group previously^25,26,46^. In brief, capillaries (internal diameter: 0.5 mm, external diameter: 1.0 mm, length: 7.5 cm) were plasma cleaned for 10 min to remove any contaminated residues and pulled using a P-2000 laser-based pipette puller (Sutter Instrument, USA) to achieve two nanopipettes under the set of a two-line pulling protocol: (1) HEAT: 775; FIL: 4; VEL: 30; DEL: 170; PUL: 80, (2) HEAT: 825; FIL: 3; VEL: 20; DEL: 145; PUL: 180. This protocol generates pores with a diameter of (21 ± 2) nm. This protocol is puller specific  and should be optimised accordingly.

### Optical setup

As reported previously^19,21^, a custom-built confocal microscope was used for all-optical measurement. Briefly, a 60x water immersion objective (1.20 NA, UPLSAPO 60XW, UIS2, Olympus) was used to introduce a 488 nm laser (Sapphire 488LP, Coherent) to illuminate the nanopipette tip and collect the generated fluorescence. The fluorescence was split into two channels (500–580 nm and 640–800 nm) using a dichroic mirror (630DCXR) and detected by two APDs (SPCM-AQR-14, PerkinElmer) respectively. A schematic and detailed description of the set-up is given in Supplementary Information (Supplementary Fig. 1). Optimal alignment of the nanopipette tip to the confocal volume was performed using an emCCD camera, where scattering was observed from the tip when high laser powers was used. Any necessary adjustments and realignment was performed to compensate for any stage drift.

### Translocation experiments and synchronised detection

Synchronised  electro-optical detection of translocation experiments was performed from the inside to the outside of the nanopipette unless reported otherwise. Analytes together with a patch/bath electrode were introduced inside the nanopipettes (*cis* chamber), and a reference electrode and buffer were placed outside (*trans* chamber). Buffer used in this work consisted of 100 mM KCl, 10 mM Tris-EDTA and 5 mM MgCl_2_ (pH = 8). For the binding assay, DNA carriers were incubated with its targets (protein/oligos) at different ratios with a final carrier concentration of 10 pM. After alignment, translocation experiments were performed by applying a potential bias using an A-M 2400 patch-clamp amplifier and corresponding current traces were recorded. Synchronised optical signals were recorded using custom written labview software.

### Data acquisition and analysis

A NI 6602 DAQ card (National Instruments) was used to record optical data while another NI-USB 6259 DAQ card was used to record the electrical data . The synchronisation of electrical and optical detection channels was triggered through a TTL pulse . The electrical signal was sampled at 70 kHz and filtered at 5 or 10 kHz using a low-pass Bessel filter. The optical photon counts were collected using APD detectors with a time resolution of 10 μs. A custom-written MATLAB code was used to analyse synchronised electrical-optical events (Supplementary Fig. 6). All mean values reported are obtained from simply taking the average of the data set, and all errors are defined as 1 standard deviation. In brief, statistics on translocations for both the optical and electrical events were obtained using the following method: 1. Track and subtract baseline signal. 2) Determine background noise level (Poisson fit) and define a cut-off threshold of 5–10 standard deviations above the mean noise level. 3) Determine the translocation times using a peak finding routine. Parameters such as peak maximum, DNA carrier maximum, total peak width, peak area are extracted. Translocation times were determined by using the FWHM of the DNA carrier signal. (4) Finally, the time stamps for both optical and electrical events were cross-correlated to obtain statistics on the percent synchronisation.

## Supplementary information

Supplementary Information

Reporting Summary

## Data Availability

The data that support the plots within this paper and other findings of this study are available from the corresponding author upon reasonable request.
