# Supplementary material for: Small molecule electro-optical binding assay using nanopores
Source: Nat Commun. 2019 Apr 17;10:1797. doi: 10.1038/s41467-019-09476-4 (PMC6470146; doi:10.1038/s41467-019-09476-4)
Supplement: Supplementary file 1 — Supplementary Information [file 41467_2019_9476_MOESM1_ESM.pdf]

Supplementary information for

Small molecule electro-optical binding assay using nanopores

Shenglin Cai et al.

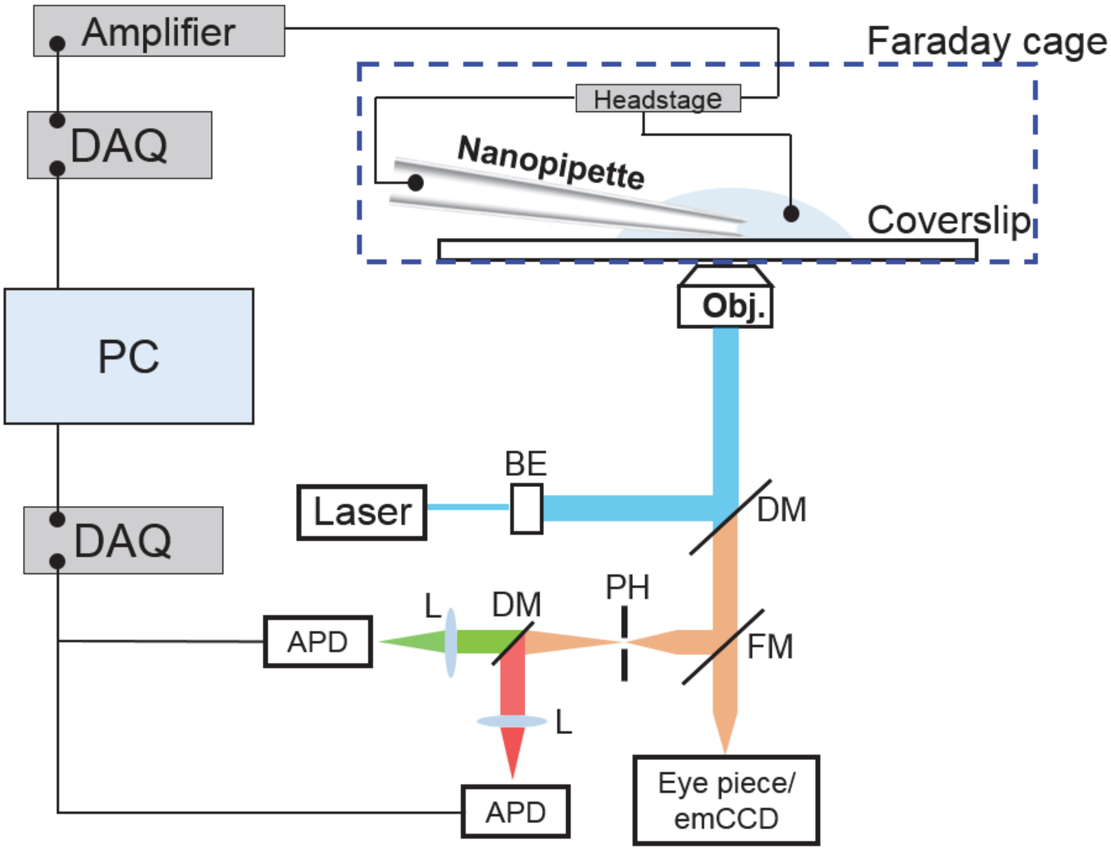

Supplementary Figure 1 | Schematic of the experimental setup. A quartz nanopipette was mounted on a coverslip and aligned to the objective of a custom-built confocal fluorescence microscope[1, 2] and adapted to incorporate a custom Faraday cage and headstage connected to an A&M 2400 Amplifier. Optical measurements were obtained using 488 nm laser excitation which was beam expanded (BE) to ensure backfilling of the objective. The laser was reflected by a dichroic mirror (DM) and introduced into the back aperture of a 60x water immersion objective (Obj.). The fluorescence from the tip of the nanopipette was collected by the same objective and passed through the same DM followed by alignment to a confocal pinhole (PH). Another DM was used to split the light into two channels (green: 500-580 nm; red: 640-800 nm) and focus the light using a lens (L) onto two avalanche photodiode detectors (APDs). The electrical and optical data were collected via two DAQ cards and triggered to record simultaneously using a custom written Labview program.

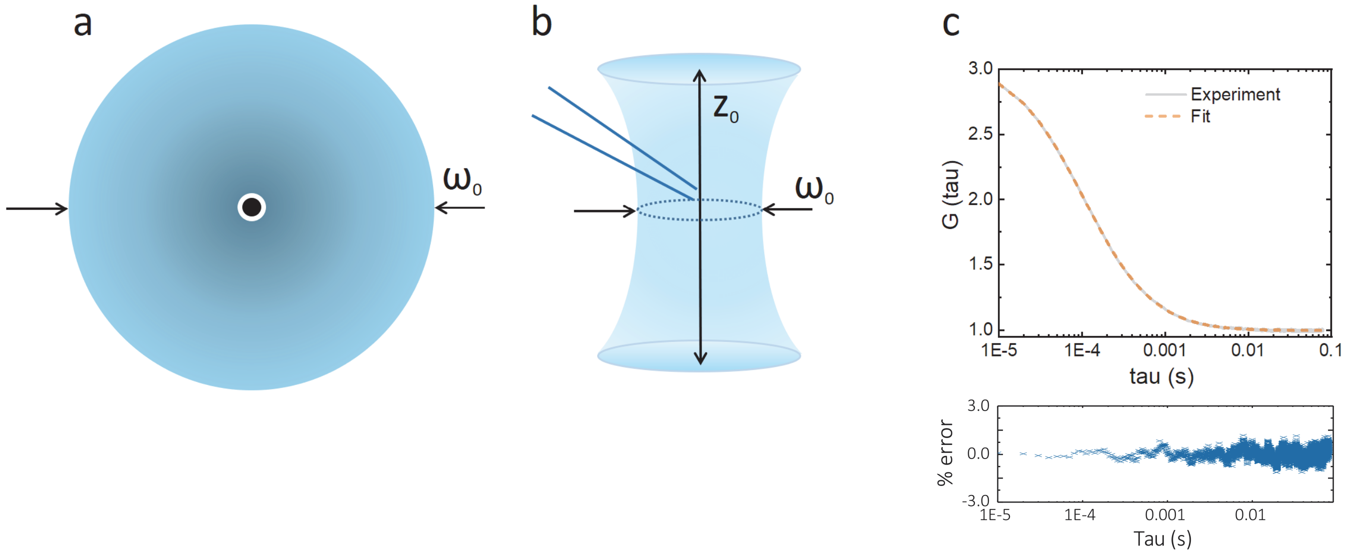

Supplementary Figure 2 | Schematic of (a) the confocal volume and a (b) cross-section of the nanopore superimposed on the confocal volume to scale. c) The confocal volume was experimentally calculated using an autocorrelation analysis using 70 pM Atto 488 freely diffusing in water. The calculated optical axis (1/e^2 waist) ω_0 is 450 ± 5 nm, and Z_0 is 1.76 ± 0.03 µm. The percent error is also plotted comparing the experimental and fit data.

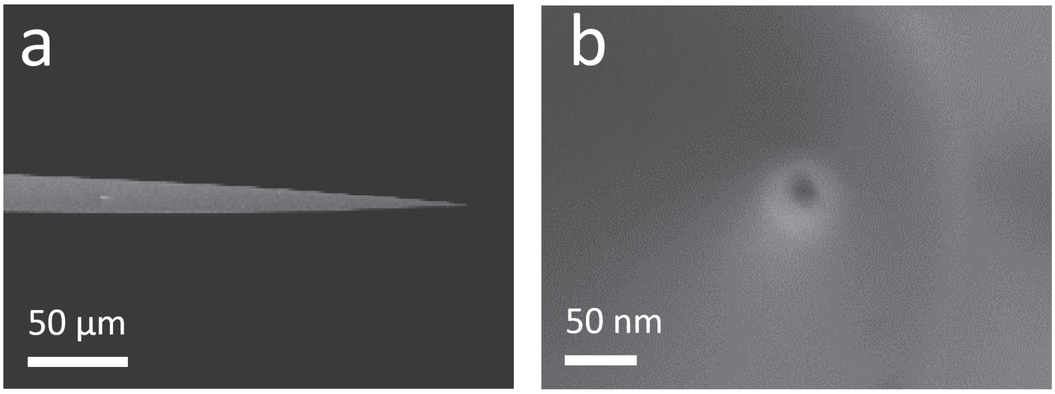

Supplementary Figure 3 | SEM images of a nanopipette. (a) SEM image showing the geometry and the taper of a quartz nanopipette. (b) SEM image of the tip with a nanopore diameter of 21 ± 2 nm.

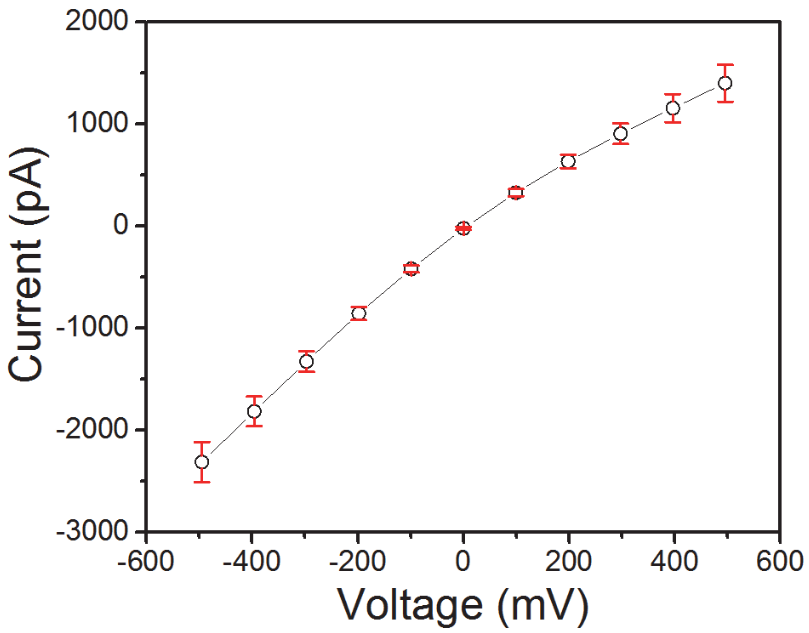

Supplementary Figure 4 | Current-voltage curves for the nanopipettes. I-V curves obtained using 100 mM KCl Tris-EDTA buffer (pH = 8.0). The conductance of the nanopipettes was estimated to be 3.7 ± 0.2 nS (n = 20).

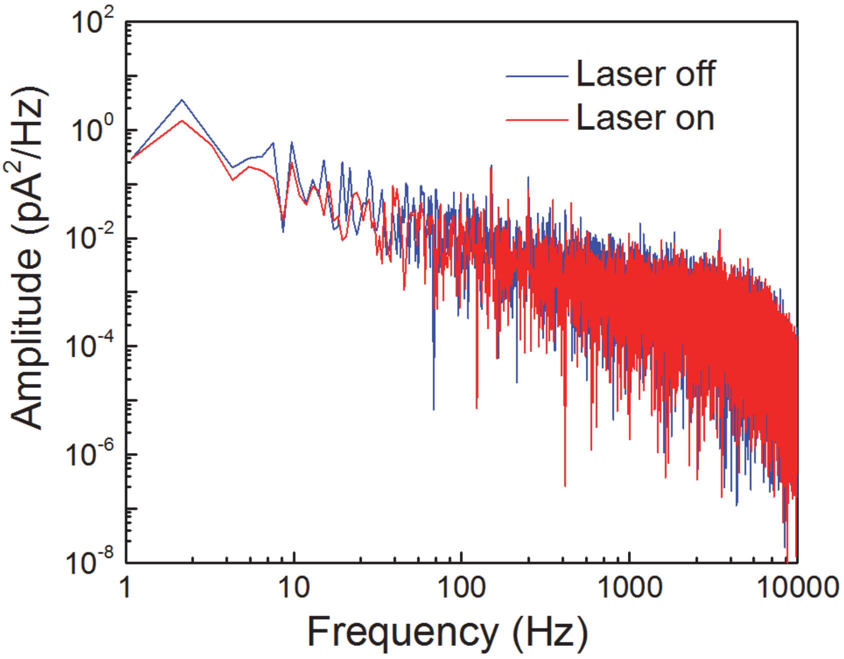

Supplementary Figure 5 | Effect of laser power on the electrical noise. Power spectrum density (PSD) plots obtained for the nanopipettes prior to and after illumination with a 488-nm laser (Power = 198 ± 6 μW). Data was obtained using an applied bias of-500 mV and a solution containing 100 mM KCl TE buffer (pH = 8.0).

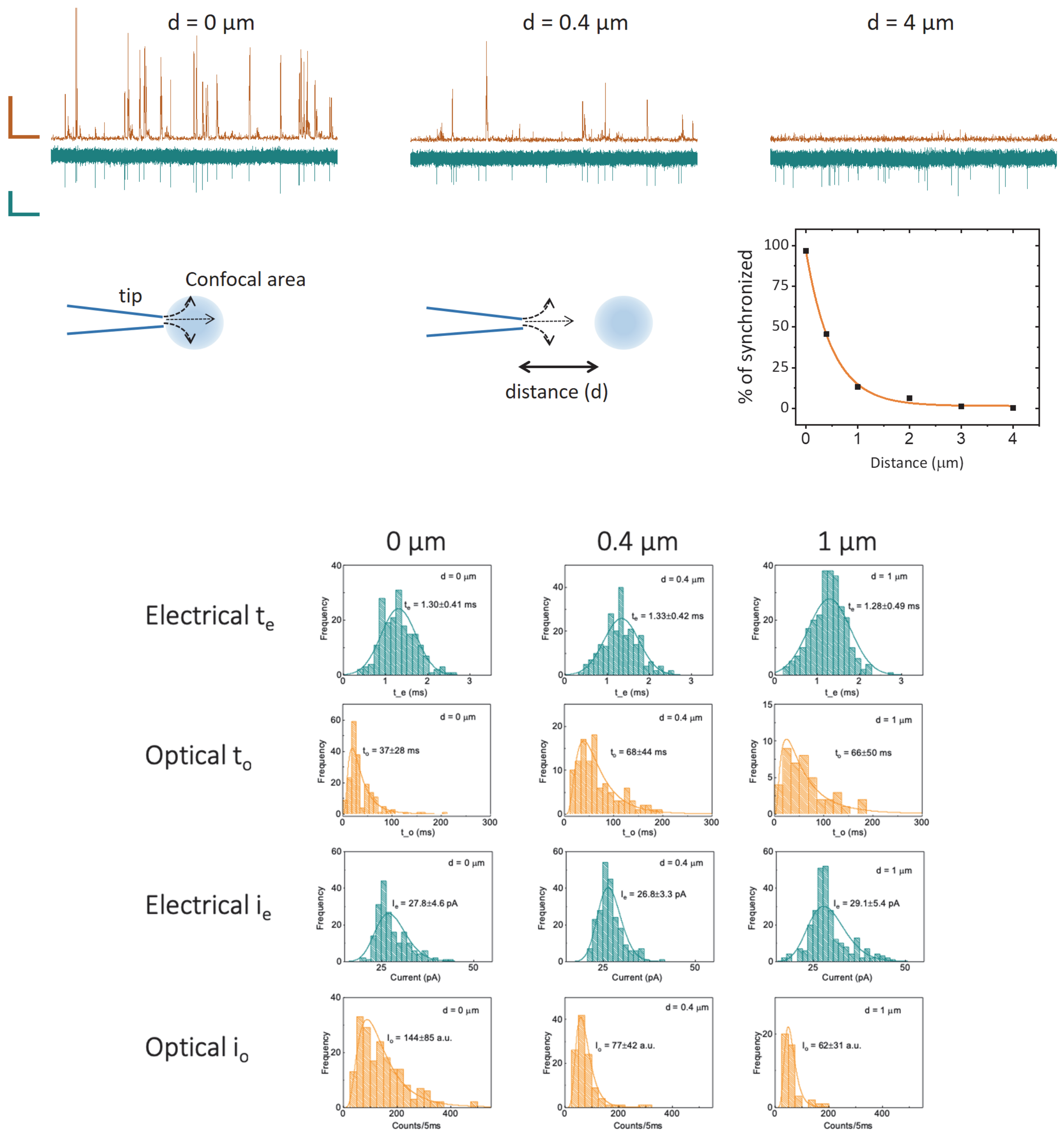

Supplementary Figure 6 | Alignment and synchronization between the nanopore and fluorescence probe volume. To assess the effect of potential drift on the per cent synchronization, the nanopipette was offset with respect to the confocal volume. A clear decrease was observed in the capture rate in the optical channel as a function of distance confirming the need minimal stage drift. Scale bars (optical, top): vertical 100 counts/5ms, horizontal 1s. (electrical, bottom): vertical 20 pA, horizontal 1s.

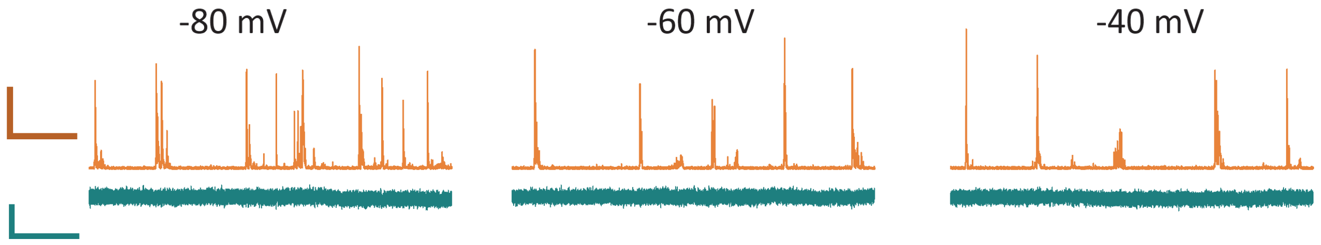

Supplementary Figure 7| Electro-optical time traces for the translocation of 5 kbp DNA-YOYO-1 at low voltages. At lower voltages (-80, -60 and -40 mV), events were only detected in the optical channel. The sample used was 5 kbp DNA-YOYO-1 (100 pM) in 100 mM KCl TE buffer (pH = 8.0). Scale bars (optical, top): vertical 200 photons, horizontal 0.5 s. (electrical, bottom): vertical 20 pA, horizontal 1 s. The resampling time for the photon time trace was 500 µs and the electrical time trace was filtered at 10 kHz. Laser power is 90 ± 3 µW.

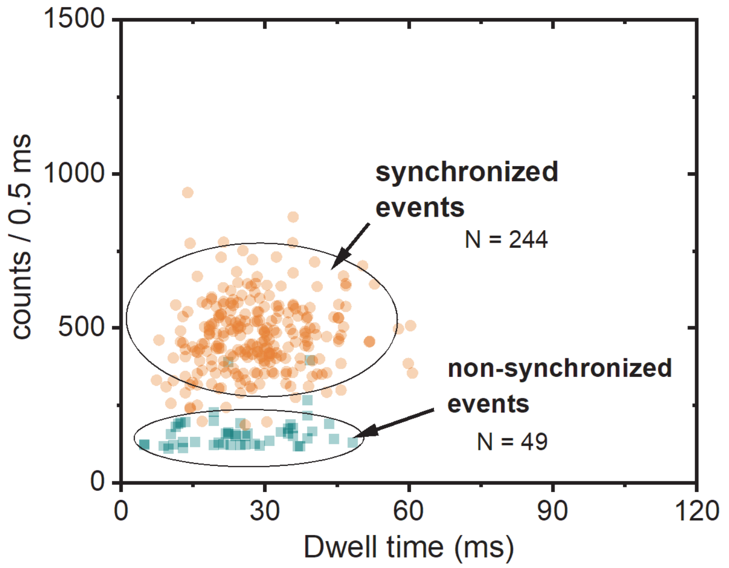

Supplementary Figure 8 | Dwell time vs Intensity scatter plot for synchronised and non-synchronised optical events. The peak height recorded for non-synchronised events (160 ± 59 counts/0.5 ms) was much lower than that for the synchronised events (470 ± 122 counts/0.5 ms). In both cases, the measurement is limited by diffusion of the molecule within the probe volume; hence the dwell times are expected to be similar. However, for synchronized events, the analyte is initially transported from inside the pipette to the outside and in the process goes through the highest intensity (Gaussian beam maximum) and in the process results in higher overall count rates. The sample used was 5 kbp DNA-YOYO-1 (100 pM) in 100 mM KCl TE buffer pH 8.0. Laser power is 90 ± 3 µW.

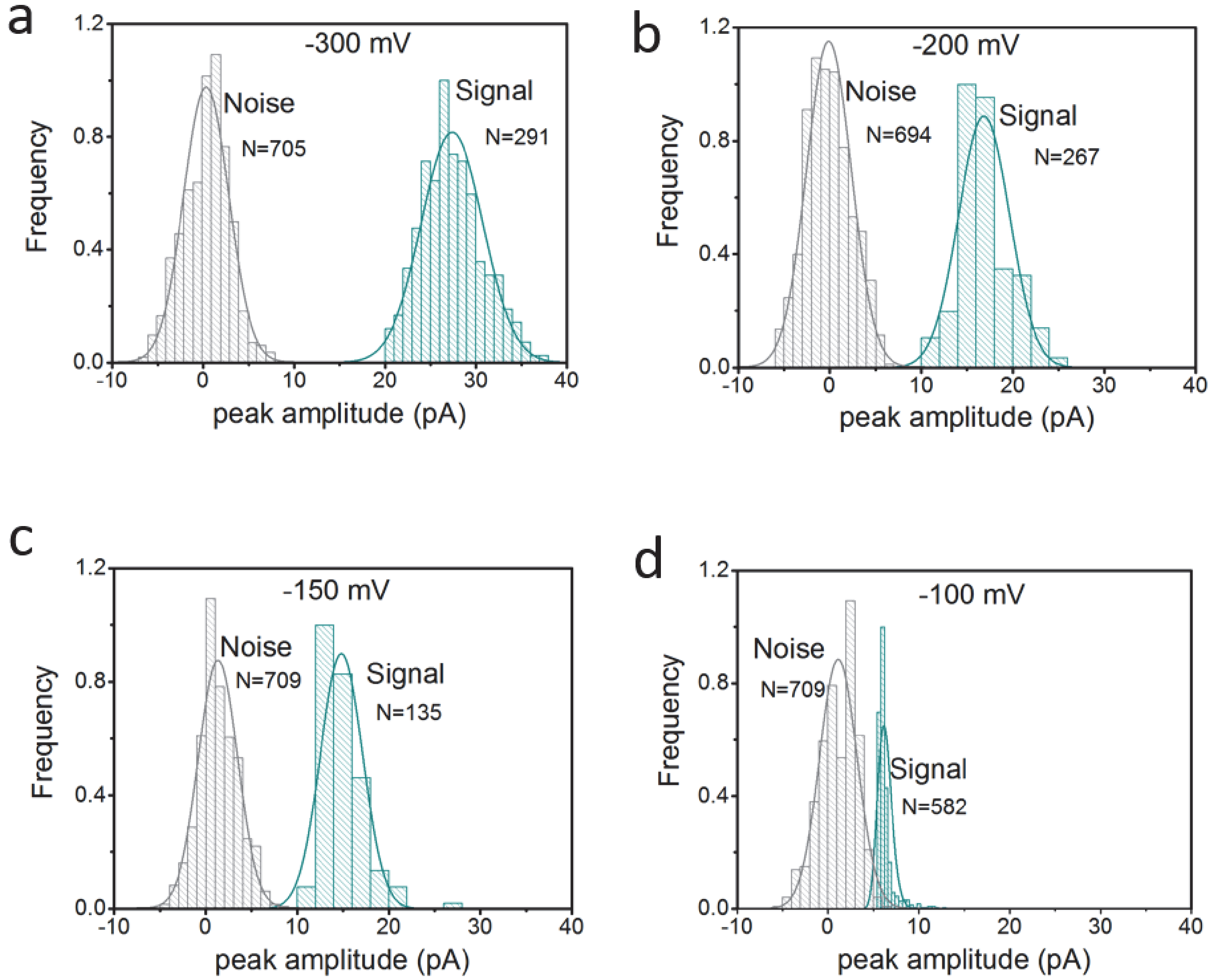

Supplementary Figure 9 | Comparison of signal and noise distributions for electrical data shown in Figure 2c. Peak amplitudes of 27.4 ± 3.4, 16.9 ± 2.8, 14.8 ± 2.3 and 6.3 ± 0.9 pA were obtained at voltages of-300, -200,-150 and-100 mV, respectively. The standard deviation in the baseline current remained relatively constant (2.5, 2.4, 2.1 and 2.0 pA, respectively). The sample used was 5 kbp DNA-YOYO-1 (100 pM) in 100 mM KCl TE buffer (pH = 8.0).

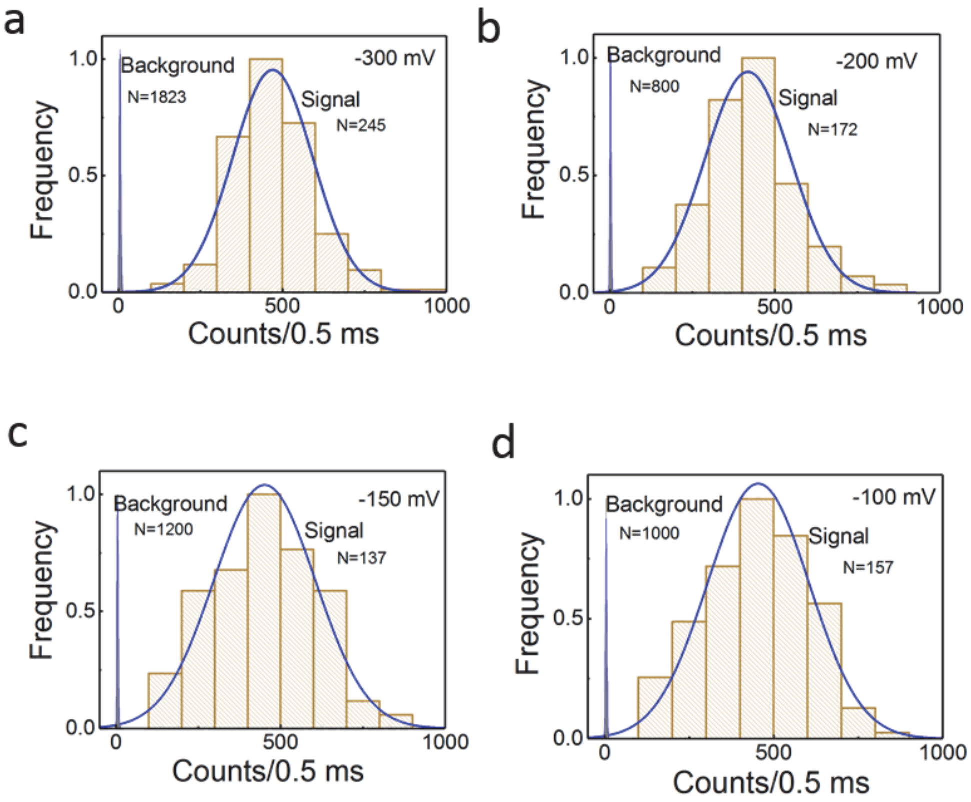

Supplementary Figure 10 | Comparison of signal and noise distributions for the optical data shown in Figure 2c. Peak amplitudes of 470 ± 122, 418 ± 130, 451 ± 154 and 454 ± 151 counts/0.5ms were obtained at voltages of -300, -200, -150 and -100 mV, respectively. The background signal remained very low and relatively constant (4.8, 4.7, 4.7, and 4.7 counts/0.5ms respectively). The sample used was 5 kbp DNA-YOYO-1 (100 pM) in 100 mM KCl TE buffer (pH = 8.0).

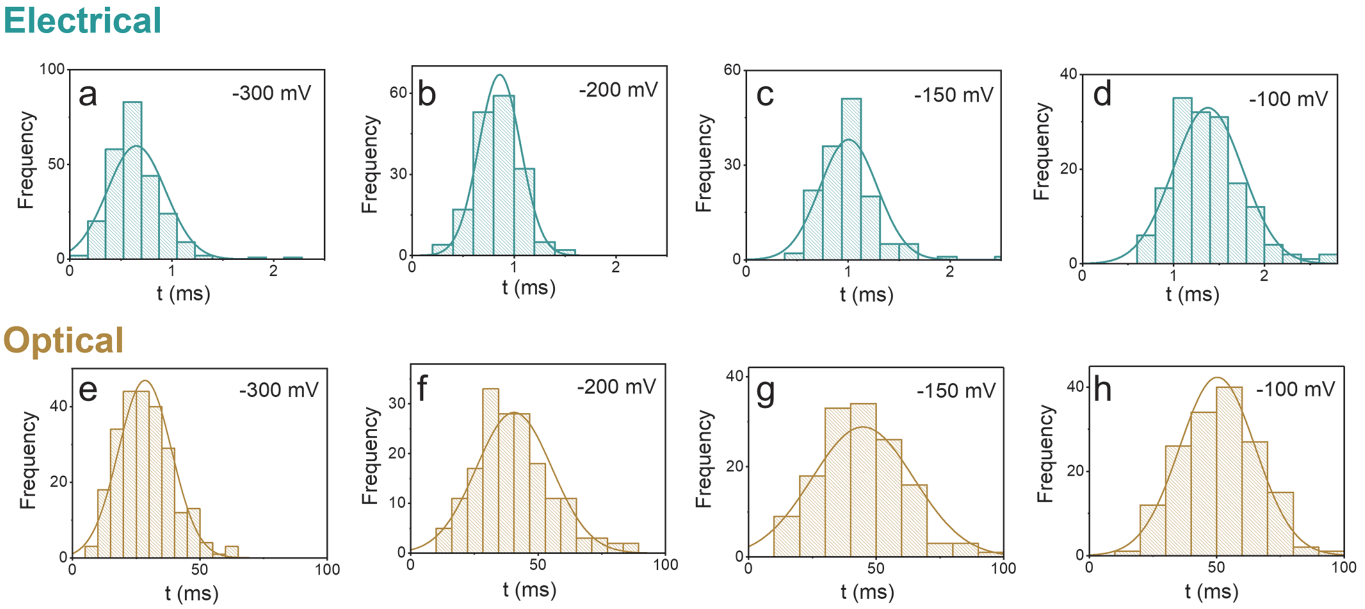

Supplementary Figure 11 | Comparison of dwell time distributions for 5 kbp DNA-YOYO-1. Mean dwell time for the electrical data **(a-d)** are 0.65 ± 0.29, 0.86 ±0.21, 1.01 ± 0.28, and 1.37 ± 0.38 ms and optical **(e-h)** data are 28.5 ± 10.4, 40.6 ± 14.8, 44.8 ± 15.1, and 50.3 ± 14.9 ms, for voltages of-300,-200,-150 and-100 mV. All data were recorded in 100 mM KCl TE buffer (pH = 8.0).

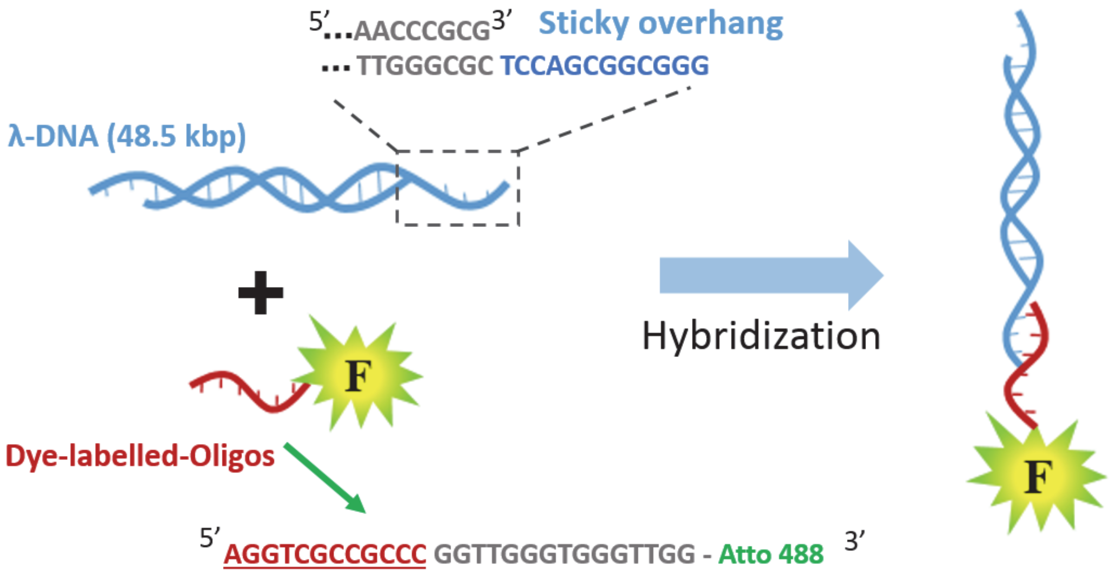

Supplementary Figure 12 | Schematic for hybridisation of λ-DNA and its complementary oligo. A 27 mer oligo (5′-AGGTCGCCGCCC GGTTGGGTGGGTTGG-Atto 488-3′) was used with the 3′ end modified to incorporate an Atto 488 label. The underlined sequence (red), was used to bind to the sticky end of the 5′ end of λ-DNA (blue, 5′-GGGCGGCGACCT- 3′).

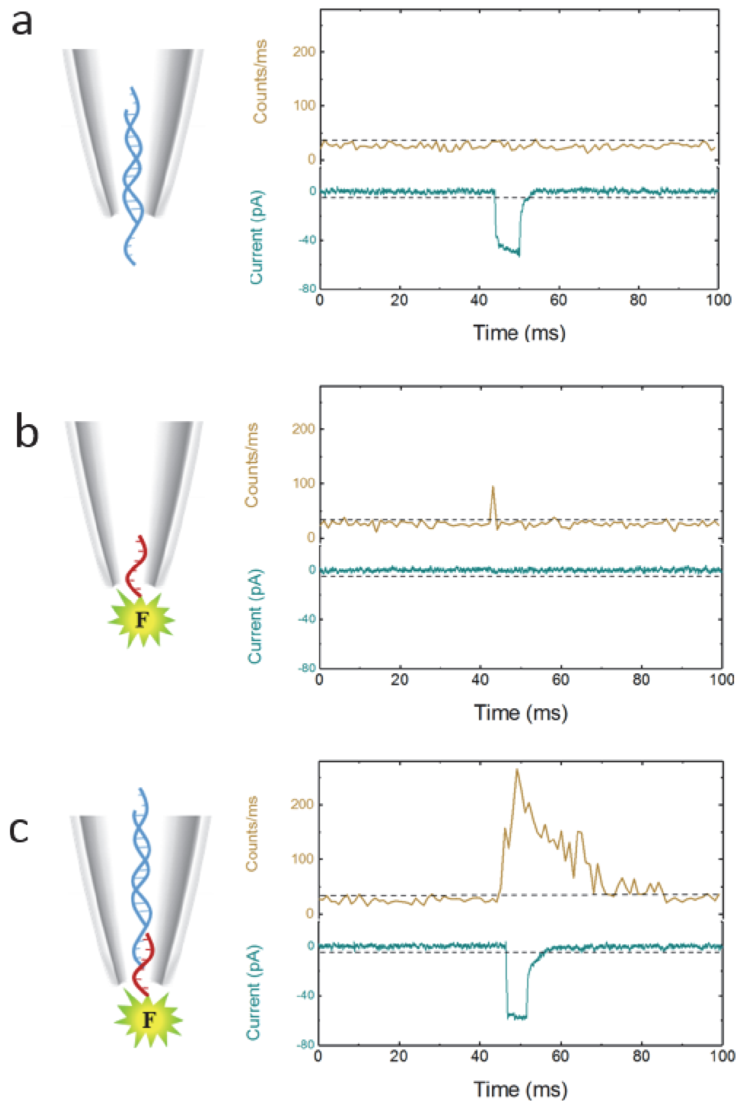

Supplementary Figure 13 | Comparison of typical electro-optical events for the translocations of (a) λ-DNA, (b) dye-oligo, and (c) λ-DNA-oligo-dye complex under a potential bias of-300 mV. The optical dwell time and total photons (peak area) detected significantly increased when the small oligos binding to the λ-DNA carrier, whereas the electrical signals (dwell time and peak amplitude) remain similar before and after the binding occurred. Laser power: 198 ± 6 μW

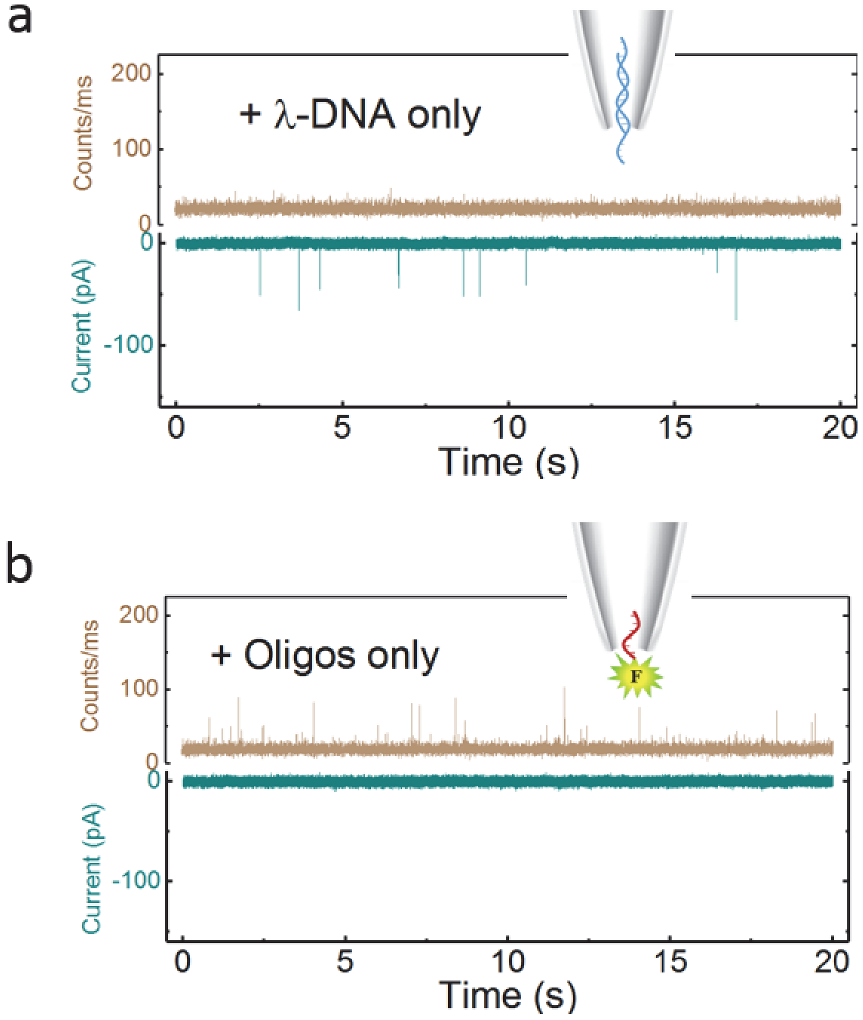

Supplementary Figure 14 | Control experiments for the translocations of λ-DNA and oligos only. Synchronised electro-optical detection was carried out using λ-DNA (10 pM) **(a)** and oligos (100 pM) **(b)**. As expected, only independent electrical or optical signals can be observed without synchronisation. Both experiments were carried out at 100 mM KCl, TE buffer (pH = 8.0) at an applied voltage of-300 mV. The resampling time for the photon time trace was 1 ms and the electrical time trace was filtered at 10 kHz. Laser power: 198 ± 6 μW.

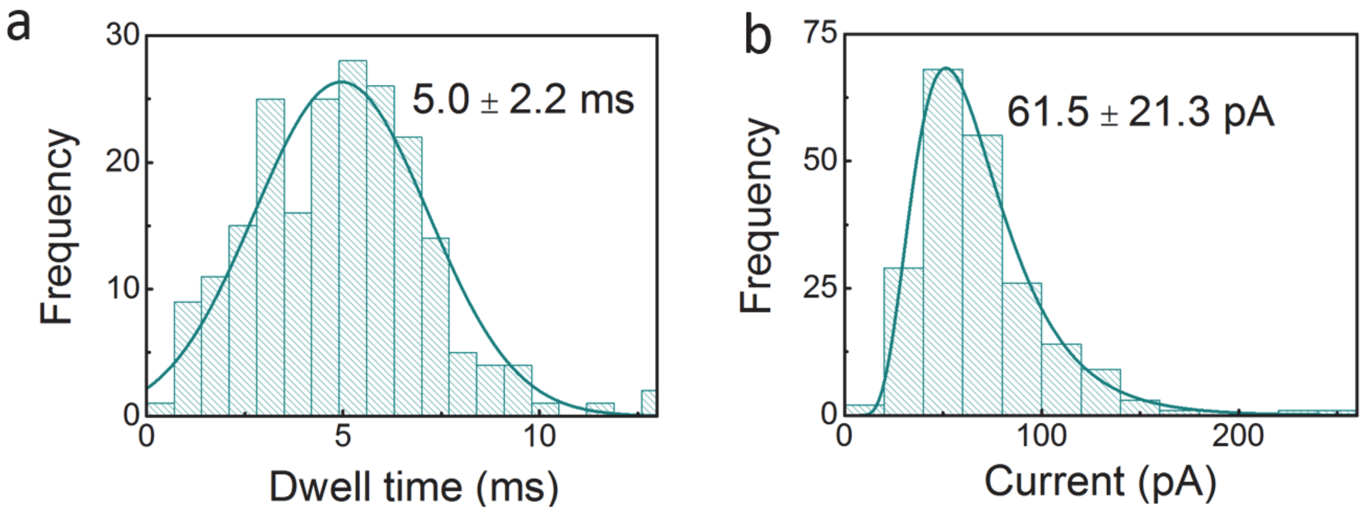

Supplementary Figure 15 | Dwell time and current amplitude distributions for the translocation of λ–DNA (control). The mean dwell time (a), and peak amplitude (b) were 5.0 ±2.2 ms and 61.5 ±21.3 pA, respectively. Data was recorded using 10 pM λ-DNA in 100 mM KCl, TE buffer (pH = 8.0) and applying a potential of-300 mV.

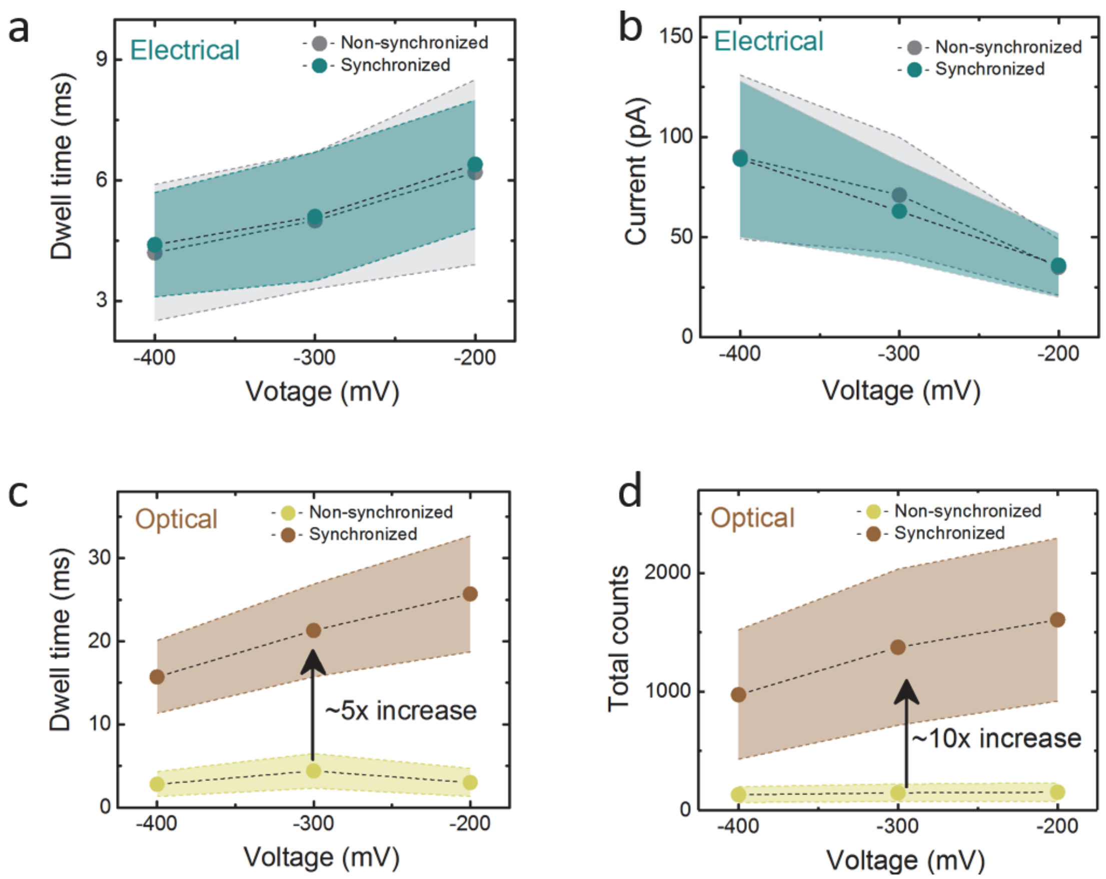

Supplementary Figure 16 | Voltage-dependence on dwell time and peak height/intensity for the translocation of the λ-DNA-oligo complex. The electrical dwell times and peak amplitudes remain constant for synchronised and non-synchronised events, **(a)** and **(b)**. Optical dwell times were approximately 5-fold slower for the synchronised events, and the total photon counts were 10-fold higher, **(c)** and **(d)**. All data were recorded using 100 mM KCl, TE buffer (pH = 8.0). The shaded area is equivalent to 1 standard deviation.

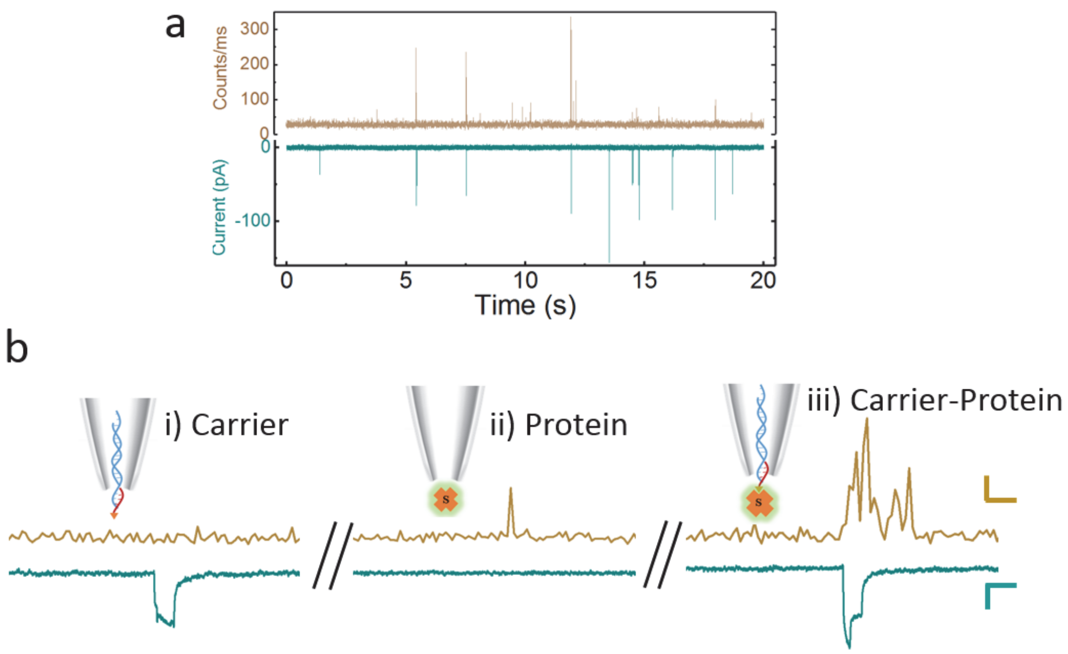

Supplementary Figure 17 | Synchronised electro-optical detection of streptavidin using biotinylated carriers. (a) A 20-second electro-optical time trace for the translocation of a biotinylated carrier (10 pM) bound to streptavidin (20 pM, Dylight 488 conjugated) at a bias of-300 mV (100 mM KCl TE buffer, pH = 8.0). Laser power 198 ± 6 µW **(b)** Three typical electro-optical signals were observed corresponding to the translocations of (i) biotinylated carrier, (ii) streptavidin, and (iii) carrier-streptavidin complex, respectively. Scale bars (optical): vertical 25 counts/ms, horizontal 10 ms. (electrical): vertical 20 pA, horizontal 10 ms. The resampling time for the photon time trace was 1 ms and the electrical time trace was filtered at 10 kHz.

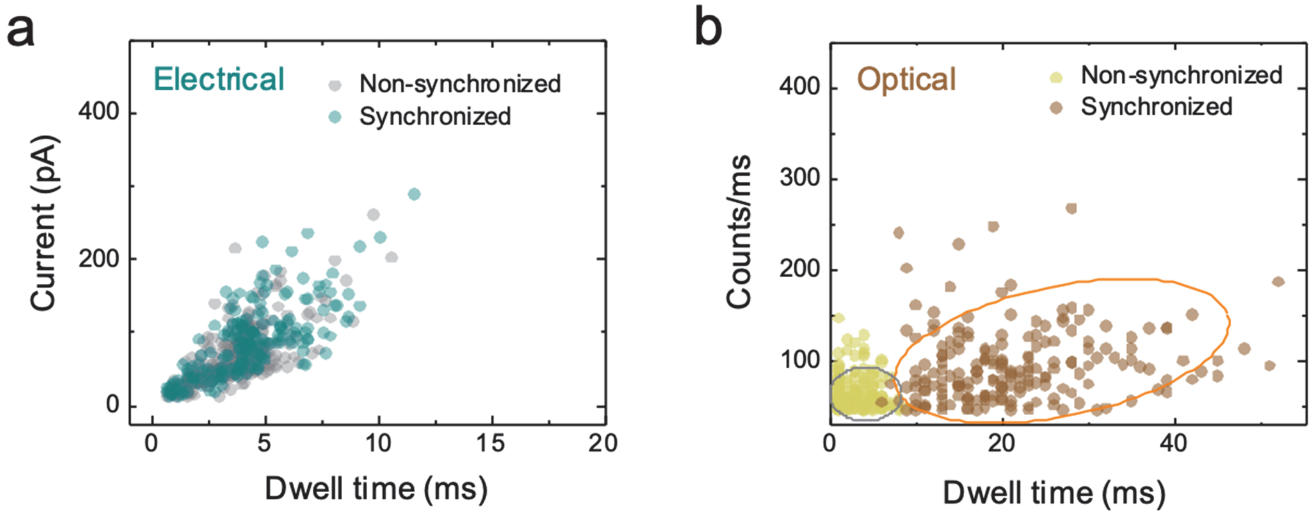

Supplementary Figure 18a | Dwell time vs intensity scatter plot for synchronised and non-synchronised events for streptavidin bound to its carrier. (a) The non-synchronised (n=274) and synchronised electrical signal (n=188) revealed nearly similar dwell times (3.9 ± 1.6 vs 4.2 ±2.1 ms) and peak amplitudes (66.8 ± 28.2 vs 76.6 ± 33.5 pA). **(b)** Synchronised optical events were significantly higher (n=188) when compared to non-synchronised events (n=156). All data were recorded using 10 pM biotinylated carriers bound to streptavidin (10 pM) at-300 mV bias in 100 mM KCl TE buffer (pH = 8.0).

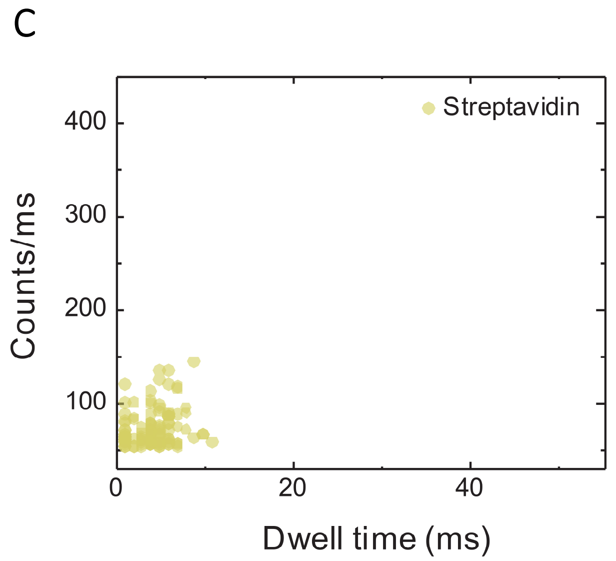

Supplementary Figure 18c | Dwell time vs intensity scatter plot for streptavidin without a carrier. Streptavidin concentration was 10 pM at translocations were performed at -300 mV bias in 100 mM KCl TE buffer (pH = 8.0). No events were observed in the electrical channel. N = 106.

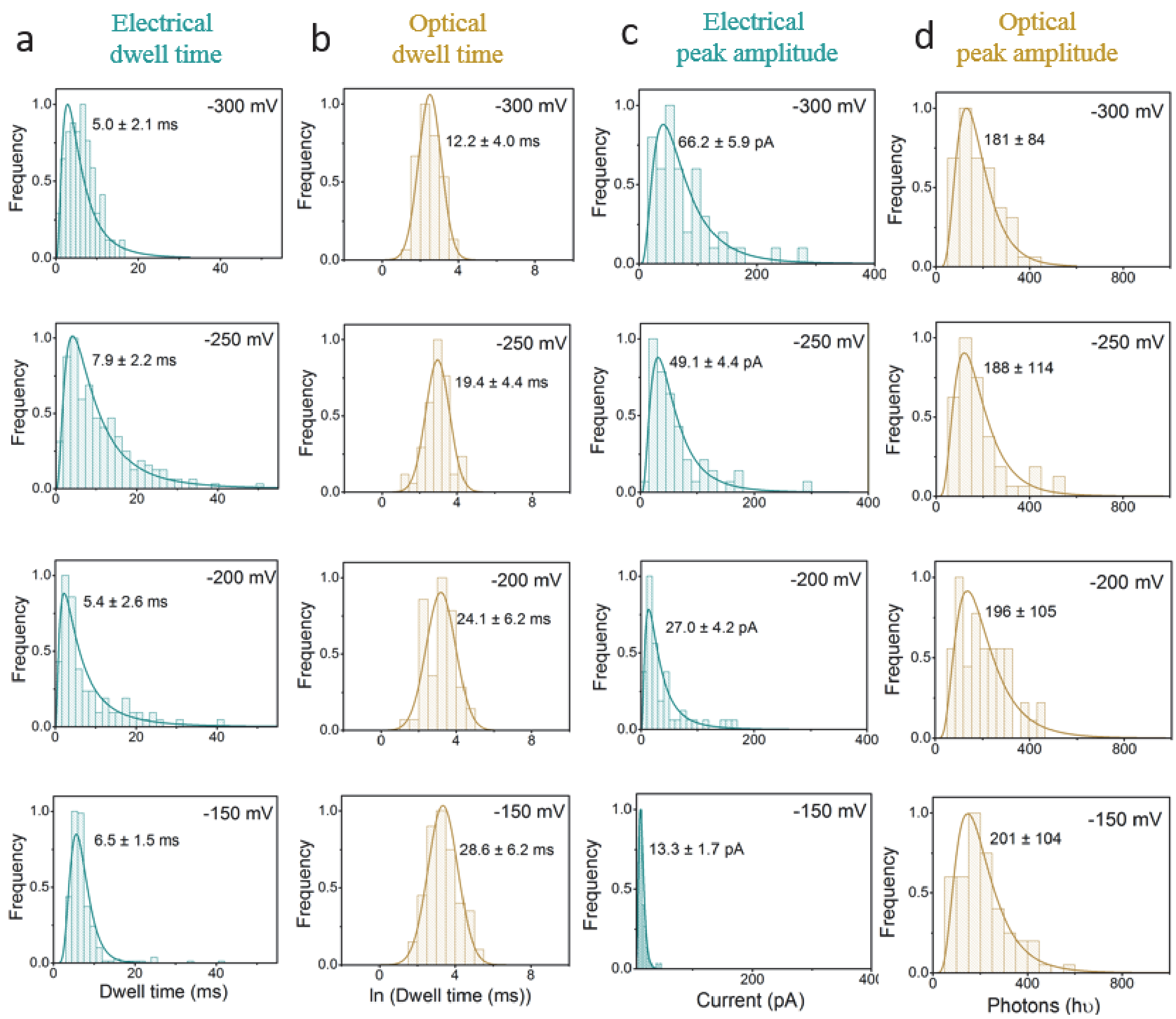

Supplementary Figure 19 | Comparison of dwell time and peak amplitude distributions for the translocation of biotinylated DNA carriers bound to streptavidin. All data were recorded in 100 mM KCl TE buffer (pH = 8.0).

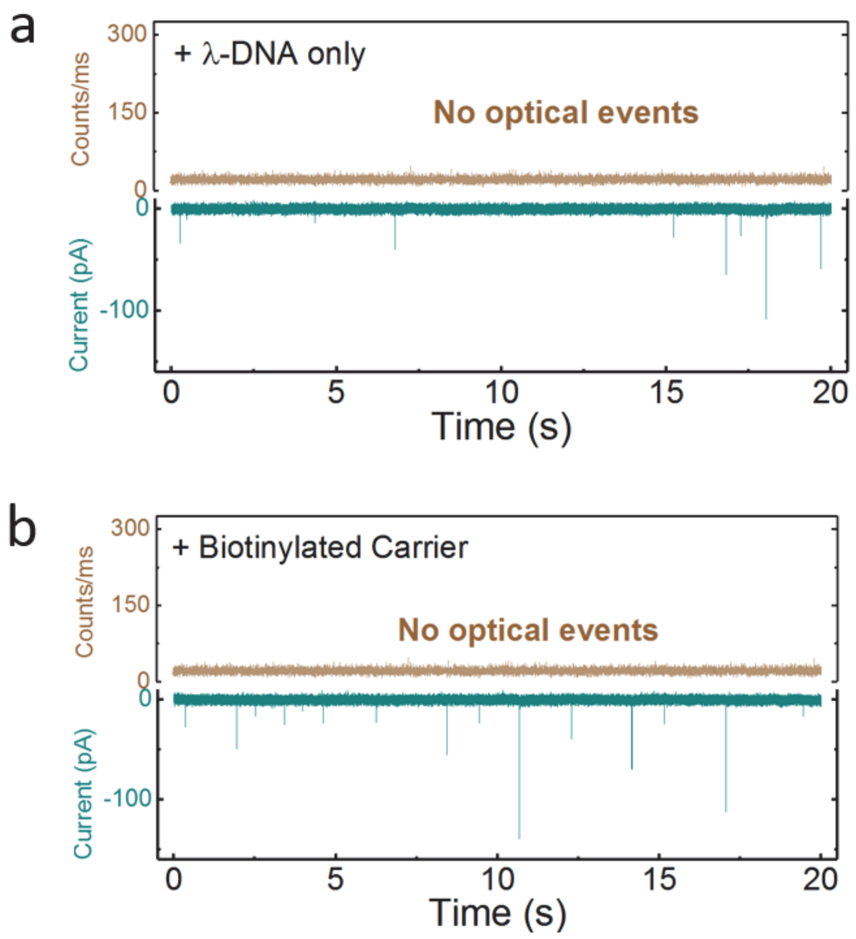

Supplementary Figure 20 | Control experiments for the translocations of λ-DNA and biotinylated carrier. Synchronised electro-optical detection was carried out for (a) λ-DNA (10 pM) (b) biotinylated carriers (10 pM). As expected, the only signal at the electrical detection channel could be observed. Both of the data were recorded in 100 mM KCl TE buffer (pH = 8.0). The resampling time for the photon time trace was 1 ms and the electrical time trace was filtered at 10 kHz. Laser power 198 ± 6 μW.

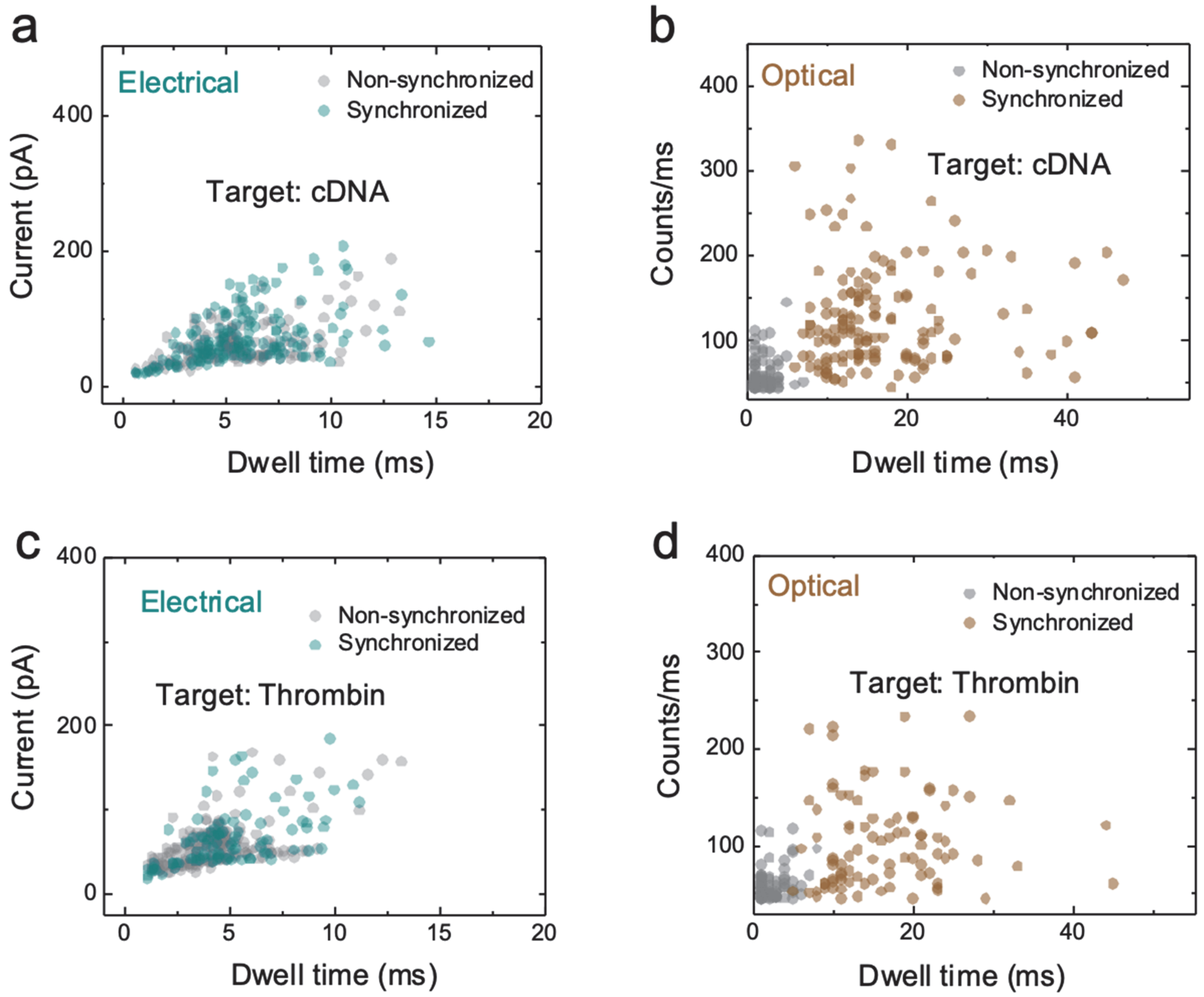

Supplementary Figure 21 | Dwell time vs Intensity scatter plot for synchronised and non-synchronised events for the MB-Carrier with cDNA / thrombin. (a) When incubating the MB-carrier (10 pM) with the cDNA or thrombin, similar electrical dwell time and current amplitude were observed for non-synchronised (5.4 ± 2.5 ms, 60.4 ± 28.3 pA, N=162) and synchronised events (5.6 ±2.6 ms, 65.4 ± 34.2 pA, N=147). (b) However, optical detection shows a clear increase in both dwell time and photon counts when comparing synchronized (N=147) and non-synchronized events (N=87). (c) With thrombin, similar dwell times and amplitudes were observed for non-synchronised (4.3 ±2.0 ms, 56.2 ± 26.6 pA, N=196) and synchronised events (4.9 ± 2.3 ms, 66.0 ± 34.8 pA, N=91. (d) Much like for cDNA, optical detection shows a clear increase in both dwell time and photon counts when comparing synchronized (N=91) and non-synchronized events (N=77). All data was recorded at-300 mV bias in 100 mM KCl TE buffer (pH = 8.0).

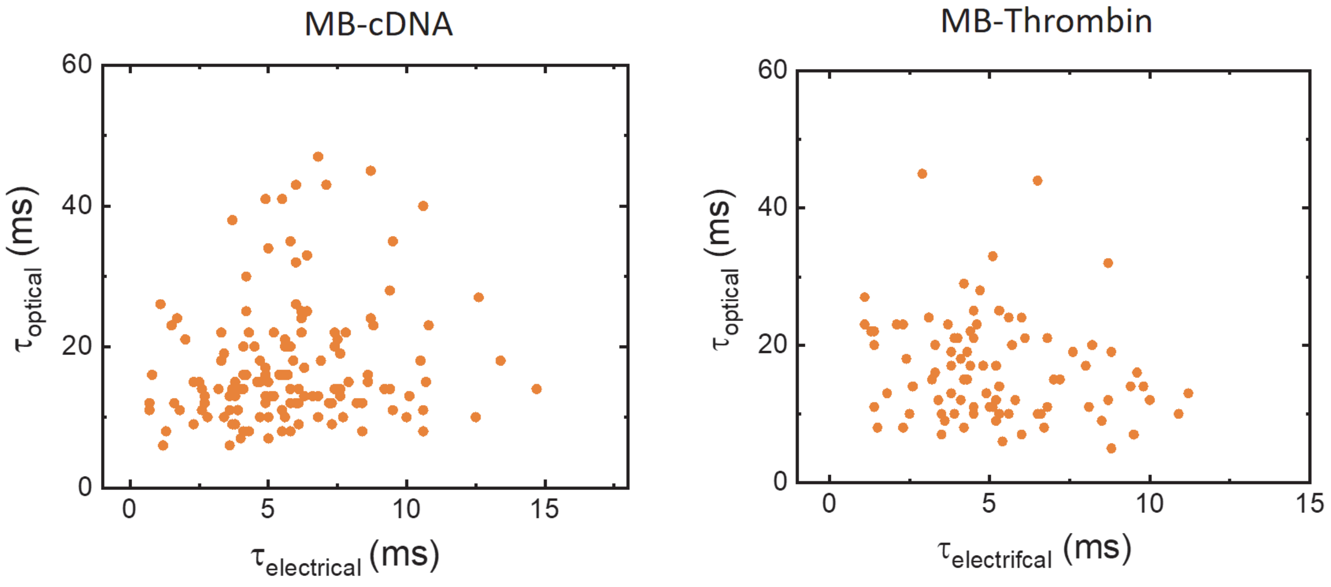

Supplementary Figure 22 | Scatter plot comparing optical and electrical dwell times for both MB-cDNA and MB-thrombin. In both cases, the carrier is attached.

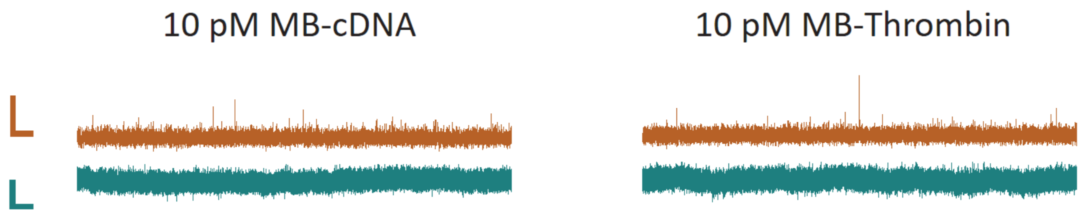

Supplementary Figure 23 | Control experiments showing intensity traces for both MB-cDNA and MB-thrombin without carriers. Scale bars (optical, top): vertical 100 counts/ms, horizontal 1s. (electrical, bottom): vertical 20 pA, horizontal 1s.

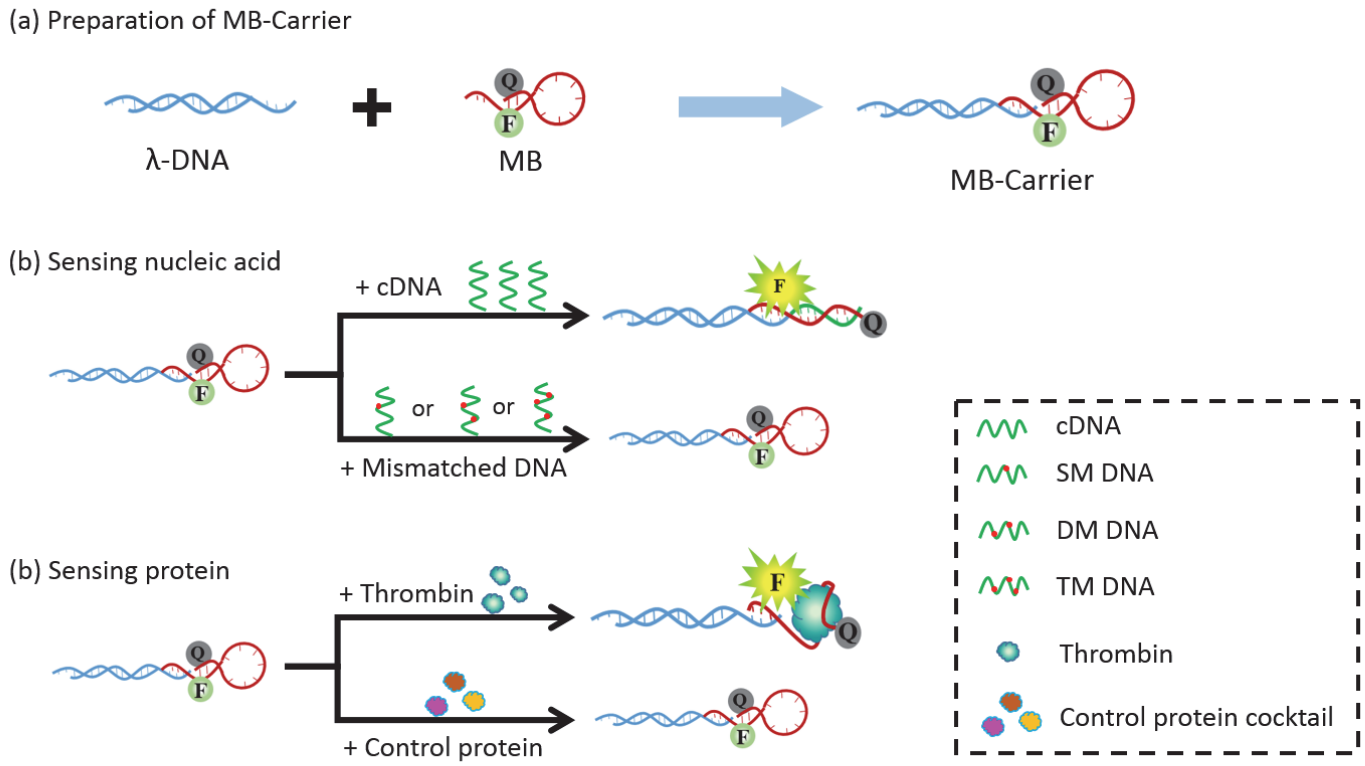

Supplementary Figure 24 | Schematic for the preparation of MB-Carrier and its binding to the nucleic acid/protein. (a) The molecular beacon was incorporated into a DNA carrier through hybridization to the 3′ end of λ-DNA (5′-GGGCGGCGACCT- 3′). The sequence of MB is as follows: 5′-AGGTCGCCGCCC-T(FAM)-***CCAAC*** GGTTGGTGTG ***GTTGG***-DABCYL-3'. The underlined bases are complementary to the 3′ end of λ-DNA. The bases in italics and bold represent the stem of the MB. The aptamer sequence incorporated into the MB targeting thrombin is shown in red. **(b)** When the MB-Carrier binding to a cDNA (15 bases, 5′-CCA ACC ACA CCA ACC-3'), the stem-loop was opened, the distance between the fluorophore probe and the quencher increases leading to restored fluorescence, while binding to single-base mismatch (SM), double-bases mismatch (DM), or triple-bases mismatch (TM) DNA, no fluorescence was observed. Sequences used are SM (5′-CCA ACC **G**CA CCA ACC-3'), DM (5′-CCA ACC **G**CA CC**G** ACC-3'), TM (5′-CCA **G**CC **G**CA CC**G** ACC-3'). The mismatched bases are bolded and underlined. **(c)** The addition of thrombin has led to the opening up of the loop to form a G-quadruplex structure and extend the distance between fluorophore and quencher, resulting in the emission of fluorescence. However, in a control protein mixture of lysozyme, trypsin, α-synuclein and insulin, no binding to the MB-carrier did not occur, and no fluorescence could be observed.

a

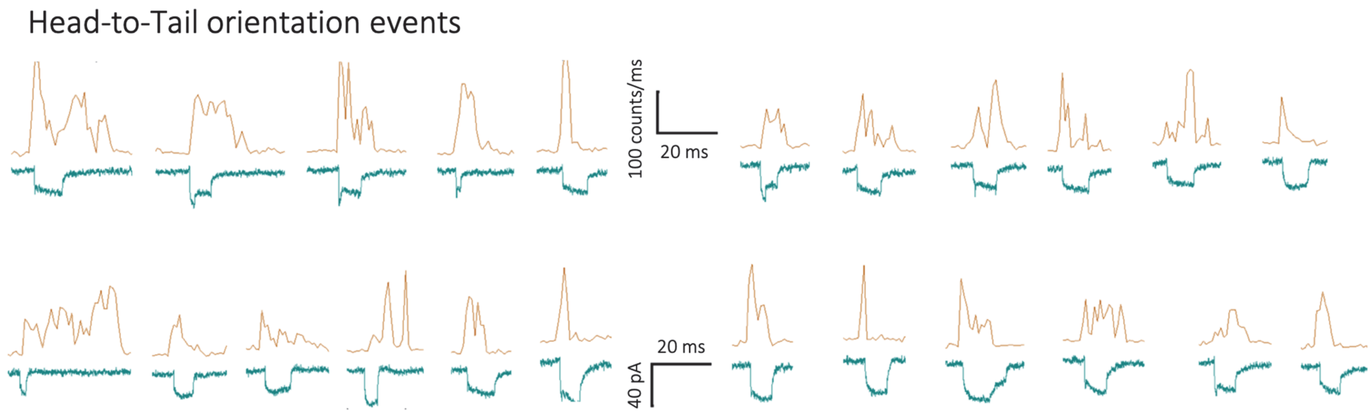

b

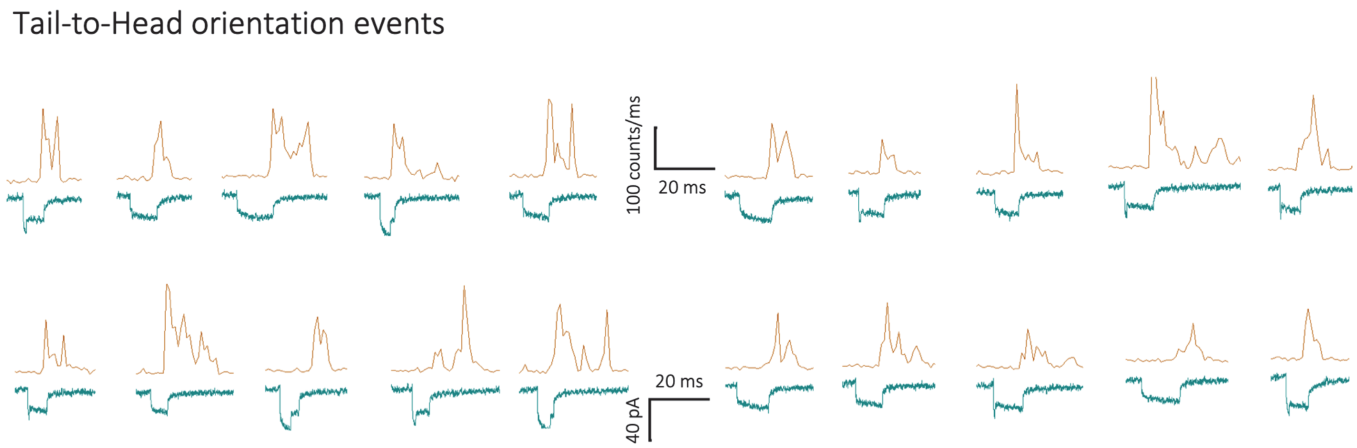

Supplementary Figure 25 | Head-to-tail and tail-to head example events used in the analysis of Figures 4c.

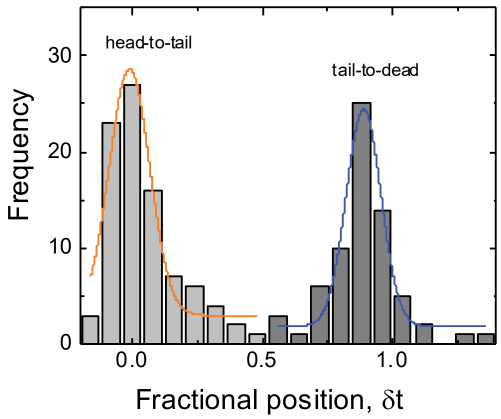

Supplementary Figure 26 | Fraction position distribution for head-to-tail and tail-to head events of MB-Carrier conjugated with cDNA for the data shown in Figure 4a-i.

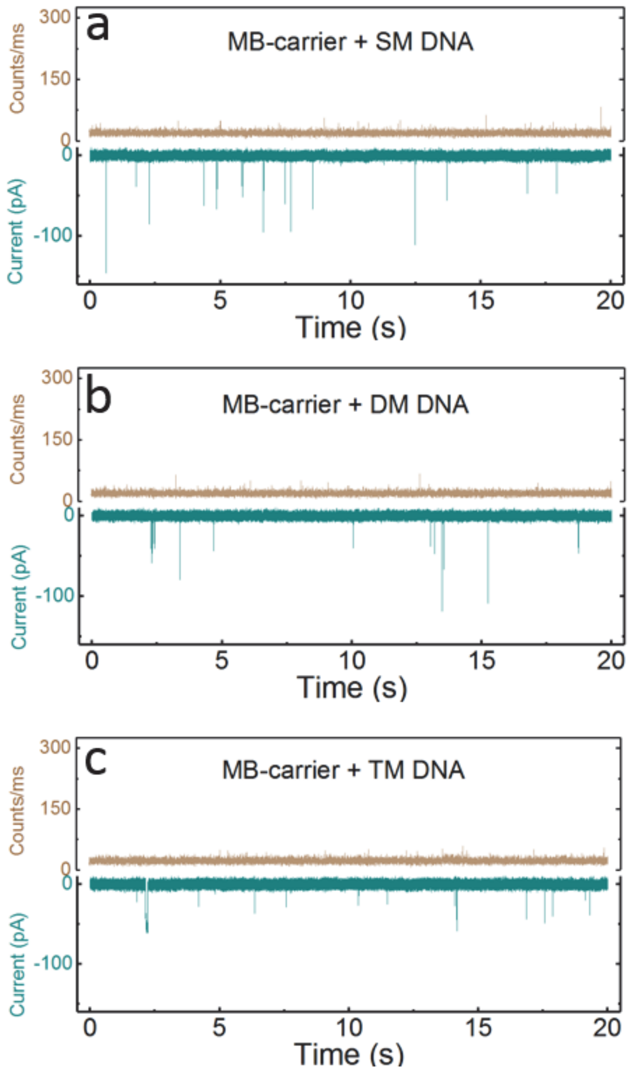

Supplementary Figure 27 | Control experiments for the MB-carrier incubating with mismatched DNA. Photon and current time traces for the translocation of 10 pM MB-Carrier incubating with (a) single-mismatch (SM), (b) double-mismatch (DM), and (c) triple-mismatch (TM) DNA, 50 pM for each. The concentration of MB-carrier was 10 pM. In these particular examples, no synchronised events were observed over the trace duration (20 s), which indicated the high selectively of the MB-carrier. All translocations were recorded at-300 mV bias using 100 mM KCl TE buffer (pH = 8). The resampling time for the photon time trace was 1 ms and the electrical time trace was filtered at 10 kHz. Laser power 198 ± 6 µW.

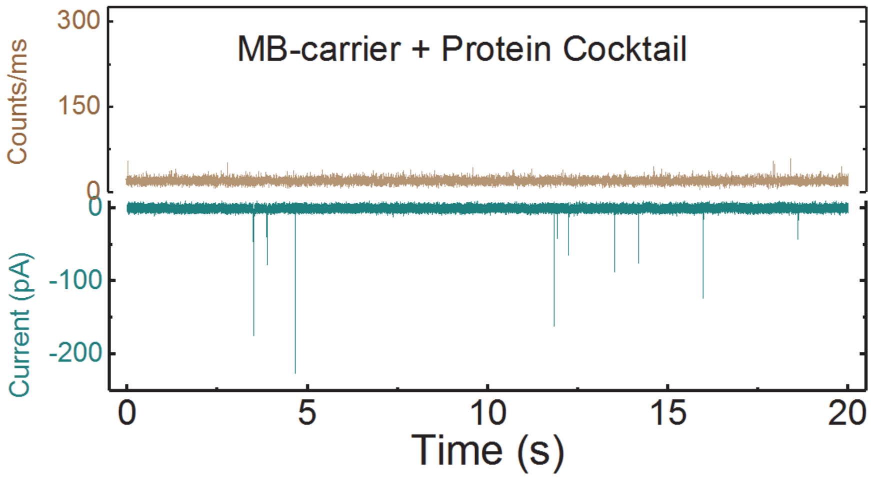

Supplementary Figure 28 | Control experiments for the MB-carrier incubating with protein mixture. Photon and current time traces for the translocation of 10 pM MB-Carrier incubating with a protein mixture (lysozyme, α-synuclein, trypsin and insulin, 10 μM for each). Same here, no synchronised events were observed indicated the selectively of the MB-carrier incorporated with TBA. Data were recorded at-300 mV bias using 100 mM KCl TE buffer (pH = 8.0). Laser power 198 ± 6 μW.

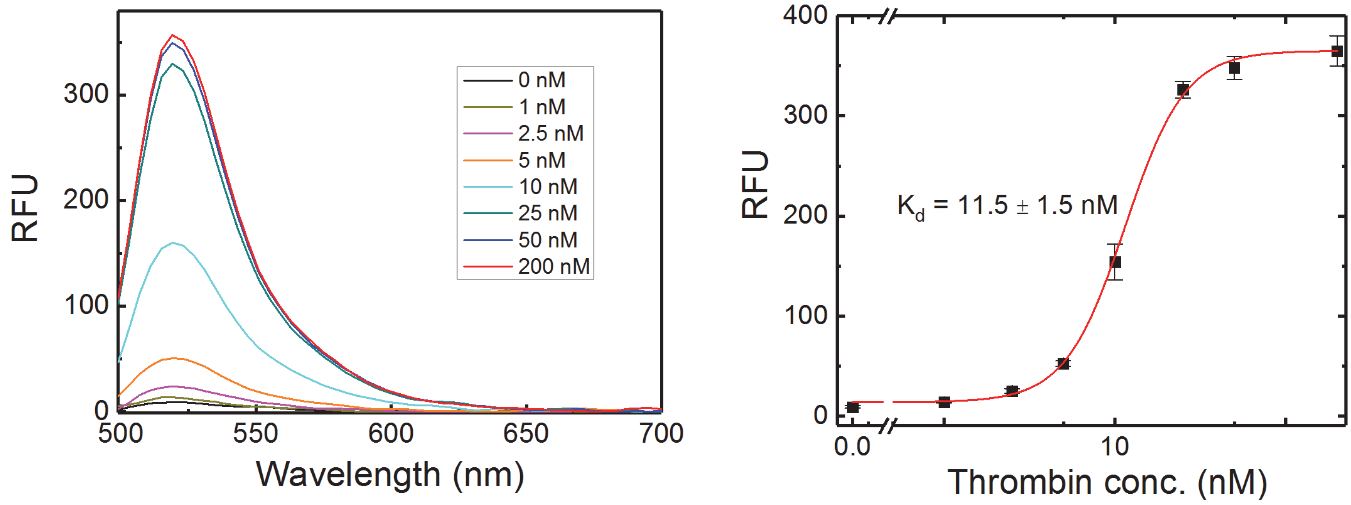

Supplementary Figure 29 | Dose-response curve for the addition of thrombin to the MB coupled carrier using bulk fluorescence methods.

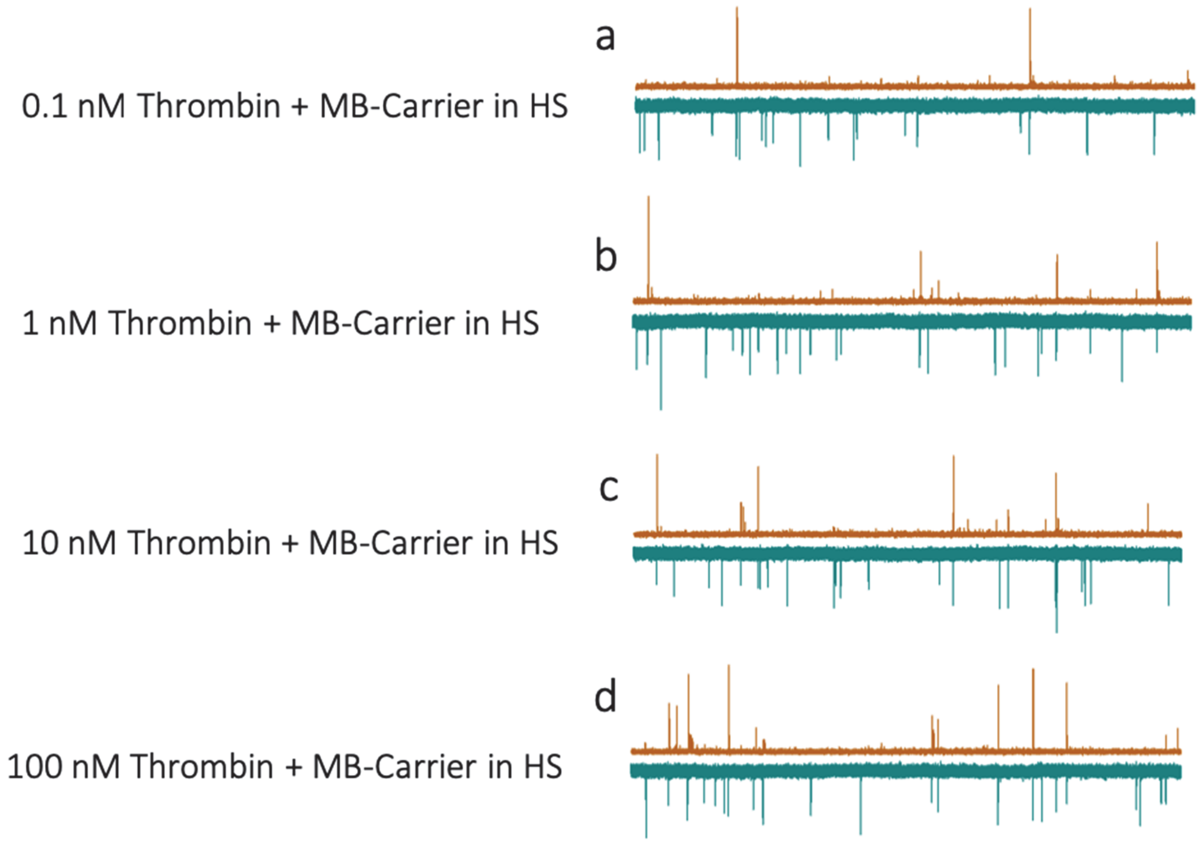

Supplementary Figure 30 | Thrombin concentration dependence in human serum. Intensity time traces were recorded at-300 mV bias using 100 mM KCl TE buffer (pH = 8.0) in 5% human serum. Laser power 193 ± 6 µW. The MB-Carrier concentration was 30 pM, and thrombin was varied between 0.1 – 100 nM.

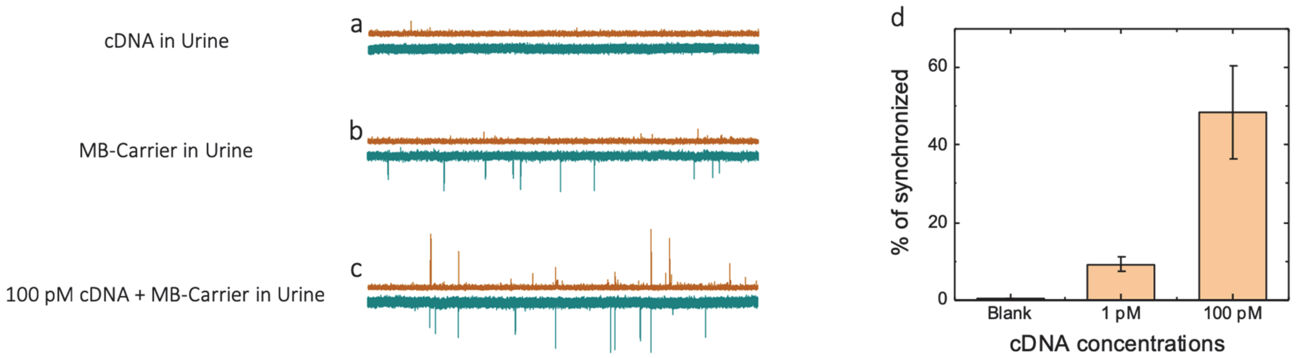

Supplementary Figure 31| cDNA concentration dependence urine. Intensity time traces were recorded at-300 mV bias using 100 mM KCl TE buffer (pH = 8.0) in 10% urine. Laser power 193 ± 6 µW. The MB-Carrier concentration was 10 pM, and cDNA was varied between 1 – 100 pM.

Supplementary Methods 1 | Sequences of DNA oligos used in this work.

Oligo sequences used for binding to λ-DNA in (Figure 3 in the main text):
5′- AGGTCGCCGCCC GGTTGGGTGGGTTGG - Atto 488-3′.
The underlined bases are complementary to the 3′ end of λ-DNA.

Biotinylated oligo sequences used for binding to λ-DNA in (Figure 3 in main text):
5′-AGG TCG CCG CCC-Biotin-3′

Molecular beacon (MB) sequence:
5′-AGG TCG CCG CCC-T(FAM)-***CCAAC*** GGTTGGTGTG ***GTTGG***-DABCYL-3'
The underlined bases are complementary to the 3′ end of λ-DNA. The bases in italics and bold represent the stem of the MB. The aptamer sequence incorporated into the MB targeting thrombin is shown in red.

Complimentary sequence (cDNA) used for binding to the MB (Figure 4)
5′-CCA ACC ACA CCA ACC-3'

Single, double, and triple base mismatched sequences used for binding to the MB (Figure 4). The mismatch underlined and in bold.

Single-mismatch: 5′-CCA ACC **G**CA CCA ACC-3'
Double-mismatch: 5′-CCA ACC **G**CA CC**G** ACC-3'
Tribble-mismatch: 5′-CCA **G**CC **G**CA CC**G** ACC-3'

Supplementary Note 1 | Calculation of the binding affinity for DNA hybridisation using Gibbs free energy.

We estimated the theoretical binding affinity, K_d, using the Van't Hoff equation,[3]

$$\Delta G = RT\ lnK_d$$

Where ΔG is the Gibbs free energy, R is the ideal gas law constant, T is the Kelvin temperature (room temperature 293 K), and the K_d is the dissociation constant. Free energy (-16.14 kcal/mol) corresponding to the complementary sequence was used and approximated using an online calculator from IDT Biophysics (http://biophysics.idtdna.com/). The calculated K_d for cDNA binding to the MB was determined to be 0.9 pM, which well agrees with that estimated in the present work (3.7 ± 0.2 pM).

Supplementary References:

1. Chansin GAT, Mulero R, Hong J, Kim MJ, deMello AJ, Edel JB. Single-Molecule Spectroscopy Using Nanoporous Membranes. Nano Letters 7, 2901-2906 (2007).

2. Pitchford WH, et al. Synchronised Optical and Electronic Detection of Biomolecules Using a Low Noise Nanopore Platform. ACS Nano 9, 1740-1748 (2015).

3. Atkins PW, De Paula J. Atkins' Physical chemistry. Oxford University Press (2006).
